# Scalable Inter‐Dielectric Engineering via Vapor‐Phase Synthesis Process for Top‐Gate MoS_2_ Thin‐Film Transistor

**DOI:** 10.1002/smll.202506282

**Published:** 2025-08-26

**Authors:** Seohak Park, Mingu Kang, Inseong Lee, Seungsun Yoo, Sejin Kim, Hyeongjin Lim, Woonggi Hong, Min Ju Kim, Cheolmin Park, Jeoungmin Ji, Seunghyup Yoo, Sung‐Yool Choi

**Affiliations:** ^1^ School of Electrical Engineering Korea Advanced Institute of Science and Technology (KAIST) 291 Daehak‐ro, Yuseong‐gu Daejeon 34141 Republic of Korea; ^2^ Graduate School of Semiconductor Technology Korea Advanced Institute of Science and Technology (KAIST) 291 Daehak‐ro, Yuseong‐gu Daejeon 34141 Republic of Korea; ^3^ Department of Electronics and Electrical Engineering Department of Convergence Semiconductor Engineering Dankook University Yongin‐si Gyeonggi‐do 16890 Republic of Korea

**Keywords:** 2D semiconductor, flexible electronics, gate‐last process, iCVD process, MoS_2_, thin‐film transistor

## Abstract

2D semiconductors are promising channel materials for next‐generation thin‐film transistors (TFTs) in Internet of Things (IoT) devices. However, their inert, dangling‐bond‐free surfaces make uniform high‐*k* dielectric integration challenging and can lead to interface defect formation. Here, a scalable inter‐dielectric engineering strategy is introduced to address this challenge, using initiated chemical vapor deposition (iCVD) to deposit an ultrathin nonpolar poly(1,3,5‐trimethyl‐1,3,5‐trivinylcyclotrisiloxane) (pV3D3) film as an interlayer between MoS_2_ and HfO_2_. This pV3D3 buffer layer forms uniformly without pinholes or clusters on MoS_2_, yielding excellent interface quality and effectively suppressing HfO_2_‐induced uncontrollable doping effect and trap formation in MoS_2_. As a result, the MoS_2_ top‐gate transistors with pV3D3/HfO_2_ dielectric exhibit nearly ideal switching characteristics, including a subthreshold swing (*SS*) of 60.9 mV dec^−1^, negligible hysteresis of ≈20 mV, and low interface trap density (*D*_it,avg_) of 8.9 × 10^10^ cm^−2 ^e^−1 ^V^−1^. Furthermore, an overlapping top‐gate structure design minimizes contact resistance, achieving an *I*_ON_/*I*_OFF_ ratio above 10^8^, a field‐effect mobility (µ_FE_) of 19.2 cm^2 ^V^−1 ^s^−1^, and minimum subthreshold swing (*SS*_min_) of 80.6 mV dec^−1^. This iCVD based inter‐dielectric method is further validated on a flexible MoS_2_ top‐gate transistors and logic circuits, demonstrating its potential for scalable and large‐area high‐performance 2D electronics.

## Graphical abstract

This study demonstrates scalable inter‐dielectric engineering for top‐gate MoS_2_ transistors by integrating ultrathin poly(1,3,5‐trimethyl‐1,3,5‐trivinylcyclotrisiloxane) (pV3D3), grown via initiated chemical vapor deposition (iCVD), as an interlayer between HfO_2_ and MoS_2_. The engineered dielectric stack enables near‐ideal subthreshold swing, high *I*_ON_/*I*_OFF_ ratio, and excellent mechanical flexibility, establishing a scalable dielectric integration scheme for high‐performance 2D functional electronics.

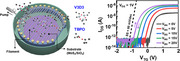

## 1 Introduction

With the advent of the Internet of Things (IoT) era, semiconductor technologies adaptable to diverse environments are rapidly gaining significance.^[^ ^1^ ^]^ However, current CMOS technology relies on rigid substrates and costly, high‐temperature processes, limiting its applicability for multifunctional uses.^[^ ^2^ ^]^ This underscores the necessity for thin‐film transistor (TFT) technologies based on novel semiconductor materials, offering lightweight, flexible, and low‐power characteristics suitable for integration into various form factors.^[^ ^3^ ^]^

The molybdenum disulfides (MoS_2_), a leading n‐type 2D semiconductor, have emerged as a strong candidate for next‐generation integrated circuits due to their superior electrical, optical, and mechanical properties.^[^ ^4^ ^]^ Its dangling bond‐free surface enables the formation of clean interfaces,^[^ ^5^ ^]^ and its ultrathin nature (≈0.7 nm) provides excellent immunity to short‐channel effects and outstanding mechanical flexibility.^[^ ^6^ ^]^ MoS_2_‐based transistors exhibit high mobility (>100 cm^2^ V^−1^ s^−1^),^[^ ^7^ ^]^ high *I*_ON_/*I*_OFF_ ratio (>10^8^), and near‐ideal subthreshold swing (*SS* ≈ 60 mV dec^−1^).^[^ ^4^, ^7^, ^8^ ^]^ Furthermore, recent advances in large‐area synthesis and low‐temperature direct growth techniques of 2D MoS_2_ enable the cost‐effective fabrication of extensive active channels on various substrates, highlighting its strong potential for large‐area flexible electronics and monolithic integrations.^[^ ^9^ ^]^

However, significant challenges still remain in fully realizing the potential of MoS_2_ for advanced functional device applications.^[^ ^10^ ^]^ A critical obstacle is the difficulty of depositing high‐quality, conformal dielectric films onto MoS_2_ via a conventional atomic layer deposition (ALD) process, primarily due to its inert and dangling bond‐free surface.^[^ ^11^ ^]^ Although notable progress has been achieved in large‐area synthesis of 2D MoS_2,_^[^ ^9^ ^]^ the absence of scalable and conformal dielectric deposition techniques continues to hinder its industrial application.

Various approaches, including surface treatment methods, van der Waals (vdW) interface engineering, and physical adsorption techniques, have been investigated to facilitate high‐*k* dielectric integration on MoS_2_. Plasma‐based surface treatment techniques, while capable of creating artificial reactive sites on MoS_2_ to facilitate ALD high‐* k* dielectrics on the channel, often introduce undesirable defects, thus limiting their effectiveness for ultrathin 2D MoS_2_.^[^ ^12^ ^]^ Conversely, vdW interface engineering methods, such as transferring 2D dielectrics (e.g., hBN, GaS) or metal‐oxide dielectrics onto 2D MoS_2_, provide excellent interface quality with MoS_2_ but suffer from limited scalability.^[^ ^13^ ^]^ Physical adsorption methods allow conformal high‐*k* dielectric deposition on 2D MoS_2_ at low temperature conditions but frequently result in compromised interface quality, introducing defects detrimental to device performance.^[^ ^14^, ^15^ ^]^ These limitations highlight the urgent need for novel dielectric deposition technologies capable of large‐area scalability without damaging the structural and electrical integrity of 2D MoS_2_.

In this study, we propose a scalable inter‐dielectric engineering approach using an initiated chemical vapor deposition (iCVD) process to deposit poly(1,3,5‐trimethyl‐1,3,5‐trivinylcyclotrisiloxane) (pV3D3) as an interlayer between ALD‐HfO_2_ and CVD‐grown MoS_2_. The solvent‐free and surface‐independent iCVD process ensures uniform and conformal deposition of ultra‐thin pV3D3 layers on MoS_2_ without pinholes or clusters, preserving the intrinsic structural and electrical properties of the underlying channel.^[^ ^16^ ^]^ The non‐polar pV3D3 inter‐dielectric, characterized by minimal hydroxyl groups, significantly enhances the interface quality by effectively reducing interface defect sites^[^ ^17^ ^]^ and suppressing HfO_2_‐induced uncontrollable charge transfer doping effect on MoS_2_.

Employing the optimized pV3D3/HfO_2_ dielectric stack, our MoS_2_ top‐gate transistors demonstrated exceptional interface quality and superior switching performance. The devices achieved a nearly ideal *SS* of 60.9 mV dec^−1^ and negligible hysteresis of ≈20 mV. The device performance was further enhanced through an overlap top‐gate configuration, where the gate electrode overlaps the source and drain regions, effectively reducing series resistance via electrostatic doping.^[^ ^18^ ^]^ This optimized structure delivered an I_ON_/I_OFF_ ratio exceeding 10^8^, field‐effect mobility (µ_FE_) of 19.2 cm^2^ V^−1^ s^−1^, and minimum *SS* (*SS*_min_) of 80.6 mV dec^−1^.

The scalability and reliability of the proposed approach were validated through the fabrication of fundamental logic circuits (NOT, NOR, NAND), demonstrating stable and reproducible device operation. Additionally, the applicability of the pV3D3 inter‐dielectric integration technique to flexible electronics was validated by electrical characterization of MoS_2_ top‐gate transistors and inverter circuits fabricated on polyimide (PI) substrates.

## 2 Results and Discussions

### 2.1 Deposition of Conformal pV3D3 Inter‐Dielectric on MoS_2_ via iCVD Process

**Figure 1:**
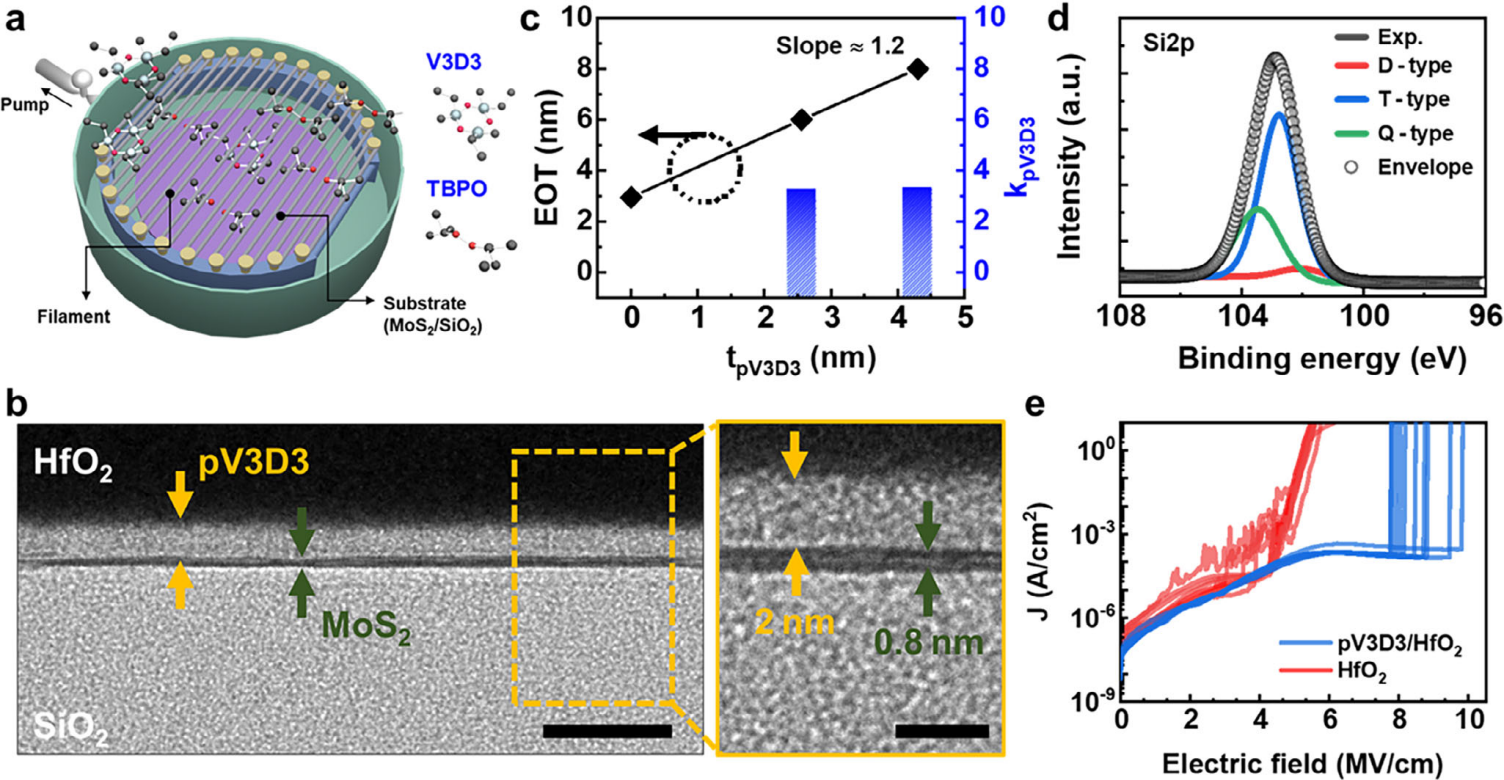
a) Schematic illustration of shower head type iCVD system. b) A cross‐sectional TEM image of the MoS_2_/pV3D3/HfO_2_ stack on a SiO_2_/Si substrate, illustrating the uniform deposition of the 2 nm pV3D3 inter‐dielectric between MoS_2_ and HfO_2_. The scale bars are 10 nm and 3 nm for the left and right images, respectively. c) Estimation of EOT and dielectric constant of pV3D3 through varying thickness of pV3D3 in pV3D3/HfO_2_ (9 nm) capacitor stacks. d) Deconvolution of Si 2p peaks in the XPS spectra of the 2 nm pV3D3 dielectric, showing di‐ (D), tri‐ (T), and quad – (Q) functional siloxane units. e) The current density as a function of the applied electric field measured for 10 different HfO_2_ and pV3D3/HfO_2_ MIM devices.

To achieve a uniform top dielectric stack on MoS_2_, the iCVD process was employed to deposit a thin and uniform pV3D3 inter‐dielectric layer, which serves as a buffer layer for subsequent deposition of ALD‐HfO_2_. Initially, monolayer MoS_2_ was synthesized on a SiO_2_ substrate via chemical vapor deposition (CVD) process and subsequently transferred onto a cleaned SiO_2_ substrate to form the active channel. Next, a 2 nm‐thick pV3D3 dielectric was deposited onto the MoS_2_ surface using a vertical showerhead‐type iCVD system (schematic illustration in **Figure** **1**a). The deposition mechanism of the pV3D3 dielectric is as follows: 1) injected 1,3,5‐trivinyl‐1,3,5‐trimethyl cyclotrisiloxane (V3D3) monomers adsorb onto the MoS_2_ surface at a cooled substrate stage temperature of 40 °C; 2) simultaneously, the injected tert‐butyl peroxide (TBPO) initiator thermally decomposes into radicals upon contact with a filament heated to 250 °C; and 3) these generated radicals react with the adsorbed V3D3 monomers on the MoS_2_ surface, forming the pV3D3 polymer through surface co‐polymerization. Finally, HfO_2_ was deposited onto the pV3D3/MoS_2_ structure via ALD, completing the pV3D3/HfO_2_ bilayer as a top dielectric stack.

Figure 1b presents a transmission electron microscopy (TEM) image demonstrating a highly conformal 2 nm‐thick pV3D3 interlayer deposited via iCVD onto the MoS_2_ surface. TEM analysis confirms that the pV3D3 layer is uniformly deposited between MoS_2_ and ALD‐HfO_2_, without observable damage to the underlying MoS_2_. The conformal deposition of pV3D3 on MoS_2_ is attributed to the surface‐independent characteristics of the iCVD process, where the low substrate temperature (40 °C) promotes physical adsorption of V3D3 monomers onto the MoS_2_ surface, enabling efficient radical‐initiated surface polymerization.^[^ ^19^ ^]^ This approach ensures uniform dielectric film formation even on surfaces with limited reactive sites, such as 2D MoS_2_.^[^ ^16^, ^20^ ^]^

To further confirm the conformality and surface integrity, atomic force microscopy (AFM) measurements were performed on MoS_2_ surfaces before and after pV3D3 deposition, as shown in Figure S1 (Supporting Information). AFM analysis reveals a root‐mean‐square (RMS) surface roughness of 0.36 nm for the pV3D3‐coated MoS_2_, closely matching the pristine MoS_2_ RMS surface roughness of 0.33 nm. This result verifies that the iCVD deposition effectively preserves the original surface morphology and structural integrity of MoS_2_. In addition, in Figure S2 (Supporting Information), we confirmed that the surface‐independent iCVD‐based deposition of pV3D3 dielectric exhibits conformality on exfoliated WSe_2_, a representative p‐type 2D semiconductor, with an RMS of ≈0.15 nm compared to the conventional ALD process. This underscores the benefit of the surface‐independent nature of iCVD in enabling conformal dielectric deposition on chemically inert 2D semiconductors, thereby supporting its extendibility to other 2D materials.

Figure 1c shows capacitance measurements of pV3D3/HfO_2_ metal (Au/Cr)‐insulator‐metal (Au/Cr) (MIM) capacitors, conducted to determine the dielectric constant of pV3D3 inter‐dielectric. Capacitors with varying pV3D3 layer thicknesses (0, 2.56, and 4.3 nm) were analyzed while maintaining a constant HfO_2_ thickness of 9 nm, allowing evaluation of the effective oxide thickness (EOT). The capacitance variation with increasing pV3D3 thickness yielded a slope of 1.2, corresponding to the dielectric constant ratio *k*_SiO2_/*k*_pV3D3_ (*k*_SiO2_ = 3.9), from which k_pV3D3_ was calculated to be 3.3. In Figure S3 (Supporting Information), to demonstrate the scalability of our pV3D3 inter‐dielectric deposition, we fabricated capacitors over a 4‐inch wafer scale. Capacitance values were extracted from nine different locations, where the dielectric stack consisted of 2 nm pV3D3 and 6 nm HfO_2_. The measured capacitance exhibited a relative standard deviation of 0.05, confirming the conformality of the pV3D3 deposited by the iCVD process.

Figure 1d presents the X‐ray photoelectron spectroscopy (XPS) results corresponding to the Si 2p peak of the deposited pV3D3 dielectric. In siloxane structures, four types of siloxane units are associated with distinct binding energies: mono‐functional (M) type at 101.4 eV, di‐functional (D) type at 102.0 eV, tri‐functional (T) type at 102.8 eV, and quad‐functional (Q) type at 103.6 eV.^[^ ^21^ ^]^ The XPS spectrum of our sample indicates that the siloxane network is predominantly composed of T‐type siloxane units.

Figure 1e shows the breakdown field (*E*_BD_) versus current density for HfO_2_‐only (11 nm) and pV3D3 (2.56 nm)/HfO_2_ (9 nm) stacked metal–insulator–metal (MIM) capacitors with Au/Cr electrodes. The pV3D3/HfO_2_ bilayer exhibits a higher average *E*_BD_ of 8.5 MV cm^−1^ compared to the HfO_2_‐only devices (4.6 MV cm^−1^). HfO_2_‐only devices exhibit variable soft breakdown behaviors due to intrinsic defect states, such as oxygen vacancies and hydroxyl groups, which could be readily formed during low‐temperature ALD (150  °C), inducing localized conduction paths that increase leakage current and promote trap‐assisted tunneling.^[^ ^22^ ^]^ In contrast, the insertion of pV3D3 effectively suppresses such soft breakdown events, owing to its intrinsically low defect density, which mitigates tunneling current through the dielectric stack.^[^ ^17b^ ^]^

To further investigate potential impacts of the iCVD‐pV3D3 deposition on the underlying MoS_2_, electrical and optical characterizations were performed as presented in Figures S4 and S5 (Supporting Information). Figure S4 (Supporting Information) compares the transfer characteristics of back‐gated MoS_2_ transistors before and after direct deposition of the 2 nm‐thick pV3D3 layer via iCVD process. The absence of a significant threshold voltage (*V*_TH_) shift or SS degradation after pV3D3 deposition indicates that the deposition process does not introduce unintended doping effects or additional defect sites in the MoS_2_ channel.

Additionally, Raman spectroscopy measurement was utilized to assess the perturbations of MoS_2_ before and after pV3D3 deposition (Figure S5, Supporting Information). The pristine MoS_2_ exhibits characteristic *A*_1g_ and *E* ^1^_2g_ peak separation of ≈19 cm^−1^, indicative of monolayer MoS_2_.^[^ ^23^ ^]^ After pV3D3 deposition, Raman spectra reveal negligible shifts in these peaks, with no notable spectral changes. Those electrical and optical results strongly suggest that the pV3D3 interlayer induces negligible doping and strain effects on the underlying MoS_2_.^[^ ^24^ ^]^

### 2.2 Analysis of the Effects of pV3D3 Inter‐Dielectric on MoS_2_ Channel

**Figure 2:**
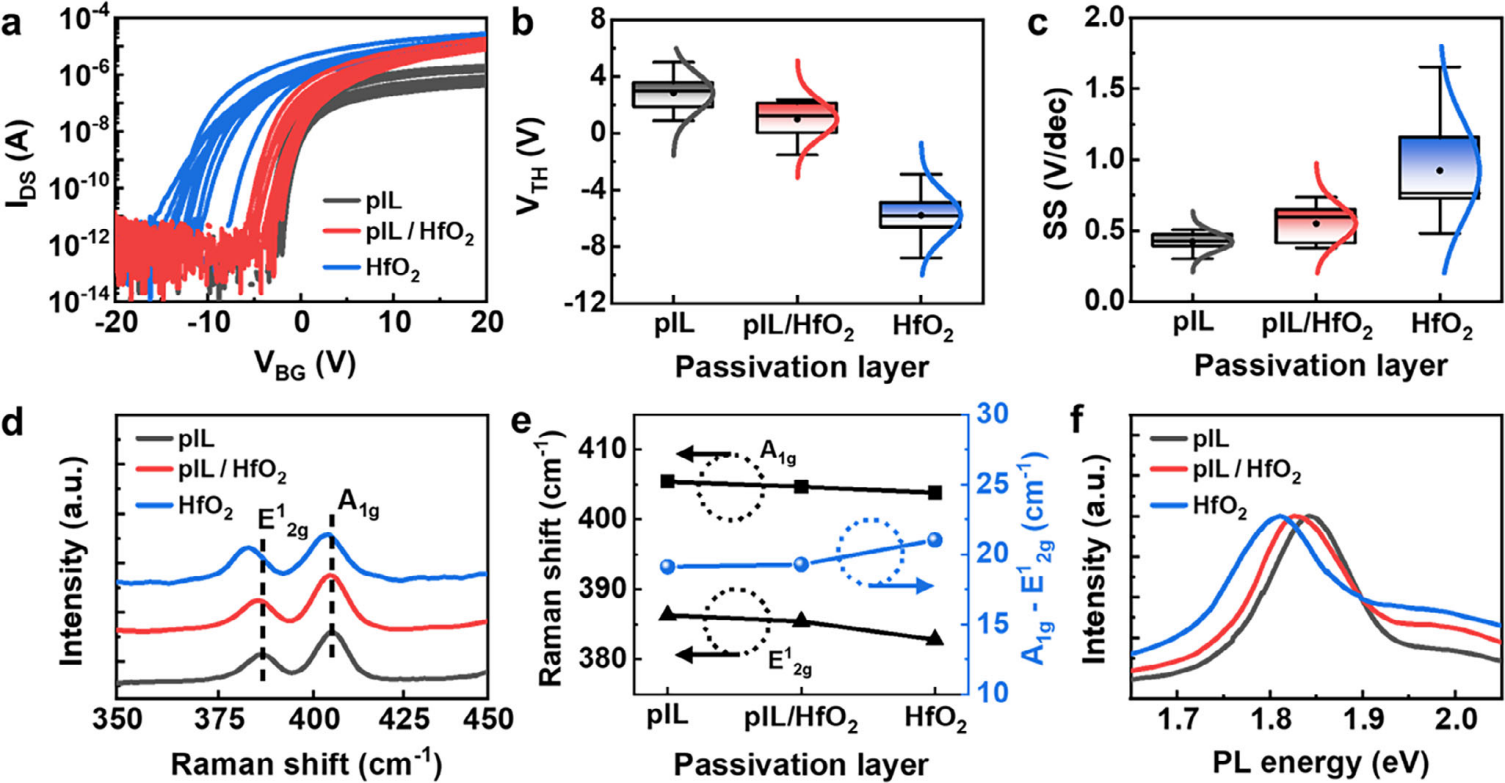
Comparison of a) transfer characteristics of back‐gate MoS_2_ transistors (channel length/width: 15 /40 µm) b) *V*_TH_ and c) *SS* distributions with three different passivation conditions: direct pIL deposition, pIL/HfO_2_ bilayer stack, and direct HfO_2_ deposition. Comparison of d) Raman spectra, e) Raman peak shifts (*A*_1g_ and *E* ^1^_2g_ peaks), and f) PL spectra for the three different passivation layers.

**Figure** **2** presents the electrical and optical characterization of back‐gated MoS_2_ transistors to examine the effect of the pV3D3 interlayer when applied as a capping layer, particularly in combination with ALD‐HfO_2_. Three different passivation schemes were evaluated: a 2 nm pV3D3 interlayer (hereafter referred to as “pIL”), direct deposition of a 6 nm ALD‐HfO_2_ layer (HfO_2_), and a bilayer configuration consisting of a 2 nm pIL followed by a 6 nm HfO_2_ layer (pIL/HfO_2_).

Direct deposition of HfO_2_ on MoS_2_ resulted in a significant negative shift in the *V*_TH_, as shown by the blue lines in Figure 2a. This shift was accompanied by a marked increase in the on‐current and degradation of *SS*. These changes are primarily attributed to strong electron charge transfer doping from defect sites in the HfO_2_ layer, along with the formation of interface defect states at the MoS_2_/HfO_2_ interface.^[^ ^14^, ^25^ ^]^ Such effects stem from the poor interfacial compatibility between ALD‐processed metal‐oxide dielectrics and 2D materials, often leading to interface degradation and undesirable electrical characteristics.^[^ ^26^ ^]^

In contrast, when a pIL was inserted prior to HfO_2_ deposition (red line in Figure 2a), the negative *V*_TH_ shift and *SS* degradation were significantly suppressed. Statistical analyses, presented in Figure 2b,c, show that the average Δ*V*_TH_ shift was reduced from ≈−8.6 V (HfO_2_) to −1.8 V (pIL/HfO_2_). Furthermore, the average *SS* in devices with direct HfO_2_ deposition was 0.92 V dec^−1^, whereas the insertion of a pIL reduced the average *SS* to 0.55 V dec^−1^, corresponding to ≈60% improvement.

The electrical characteristics presented above demonstrate that the inserted pIL layer between HfO_2_ and MoS_2_ functions not only as a uniform buffer layer that facilitates conformal HfO_2_ deposition while suppressing the formation of additional interfacial defects, but also as an effective tunneling barrier that inhibits electron transfer from defect sites—such as oxygen vacancies (V_O_) in the HfO_2_ layer—to the MoS_2_ channel, thereby enabling more precise control over the *V*_TH_ of the MoS_2_ transistors.^[^ ^27^ ^]^

To further validate the role of the pIL in mitigating the adverse effects of HfO_2_ on MoS_2_, comprehensive Raman and photoluminescence (PL) analyses were conducted. As shown in Figure S5 (Supporting Information), no significant changes were observed in the Raman peaks (*E* ^1^_2g_ and *A*_1g_) before and after the deposition of the pIL. However, direct deposition of HfO_2_, as illustrated in Figure 2d,e, induced notable red‐shifts in the *A*_1g_ and *E* ^1^_2g_ peaks by ≈1.56 and 3.47 cm^−1^, respectively. These shifts are indicative of significant electron doping and tensile strain effects induced by HfO_2_ on the MoS_2_ channel.^[^ ^24^, ^28^ ^]^ In contrast, the insertion of the pIL prior to HfO_2_ deposition effectively reduced the red‐shifts of the *A*_1g_ and *E* ^1^_2g_ peaks to 0.70 and 0.86 cm^−1^, respectively, demonstrating its capability to suppress unintended electron doping and strain effects induced from HfO_2_.

Similarly, normalized PL spectra shown in Figure 2f reveal a reduction in the peak red‐shift from 32 to 13 meV when the pIL is inserted, further confirming that the pIL effectively mitigates the electron doping effect induced by HfO_2_.^[^ ^29^ ^]^ Additionally, the normalized intensity of the B‐exciton peak near 2.0 eV, commonly associated with defect states in MoS_2_, was significantly reduced upon pIL insertion.^[^ ^30^ ^]^ This indicates that the pIL not only prevents electron doping effects but also inhibits the formation of defect states introduced by the HfO_2_ overlayer.^[^ ^31^ ^]^

Overall, those electrical and optical results clearly demonstrate that the pIL functions as an effective inter‐dielectric layer, suppressing unintentional doping, tensile strain, and defect generation in the MoS_2_ channel during ALD‐HfO_2_ integration. These advantages are particularly critical for enabling low‐power MoS_2_‐based electronic devices, where minimizing negative *V*_TH_ shift while maintaining steep switching characteristics is essential for optimal performance.^[^ ^32^ ^]^

### 2.3 Electrical Characterization of MoS_2_ Top‐Gate Transistors with pV3D3 Inter‐Dielectric Engineering

**Figure 3:**
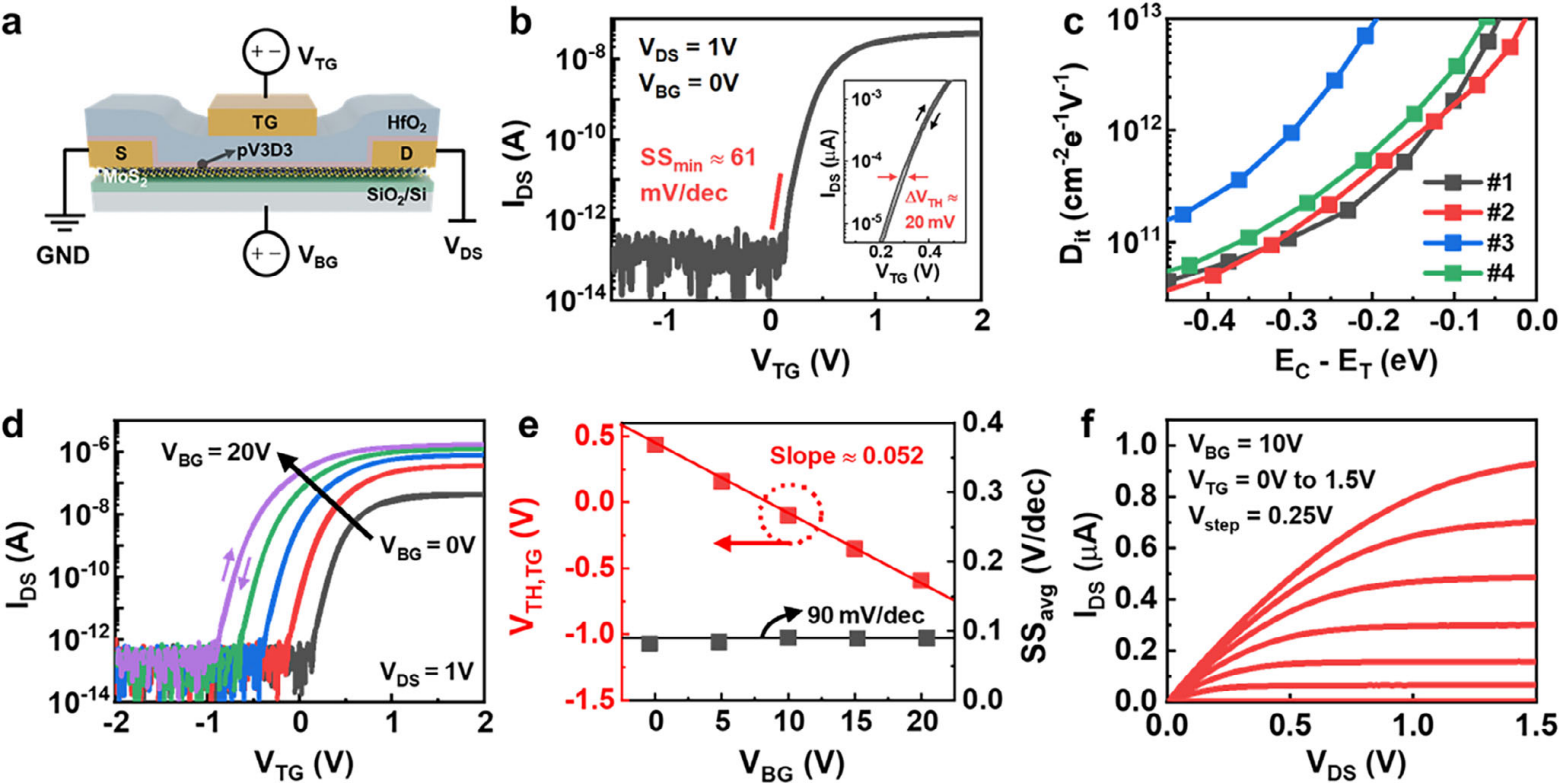
a) Schematic illustration of the underlap‐TG MoS_2_ transistor with pIL/HfO_2_ gate insulator stack. b) Transfer characteristic of the MoS_2_ top‐gate transistor at *V*_BG_ of 0 V where the inset figure shows the hysteresis property of the device. c) Estimated *D*_it_ as a function of energy for four different MIS capacitors. d) Dual‐sweep transfer characteristics under various *V*_BG_ ranging from 0 V to 20 V with a *V*_BG_ step of 5 V. e) The *V*_TH_ and 4‐order average *SS* value of the top‐gate transistor as a function of *V*_BG_. f) Output characteristic as a function of *V*_TG_ at *V*_BG_ = 10 V.

To evaluate the feasibility of pIL as an inter‐dielectric layer for a top‐gate insulator on MoS_2_ channel, we fabricated a top‐gate MoS_2_ transistor using a pIL/HfO_2_ bilayer stacked gate insulator as schematically illustrated in **Figure** **3**a. First, CVD‐grown MoS_2_, transferred onto a SiO_2_ substrate, was patterned as the active layer, and then Cr/Au source and drain electrodes were deposited via thermal evaporation, defining a channel length and width of 16.5 and 25 µm, respectively. Subsequently, a pIL layer with a thickness of ≈2 nm was deposited via iCVD as the inter‐dielectric layer, followed by the deposition of 6 nm HfO_2_ dielectric via ALD to complete the top‐gate insulator stack. Finally, the Cr/Au top‐gate electrode was defined without overlapping between the gate and source/drain metal region (underlap‐TG), with the access region between the gate and source/drain electrodes with 8 µm, as shown in the optical microscope (OM) image in Figure S6a (Supporting Information). A detailed fabrication process is illustrated in the Experimental section.

Figure 3b represents the transfer characteristic of the MoS_2_ transistor with underlap‐TG structure as a function of top‐gate voltage (*V*_TG_) under a drain to source voltage (*V*_TG_) of 1 V. The device shows *I*_ON_/*I*_OFF_ of ≈2 × 10^6^, near ideal switching property with *SS* value of 60.9 mV dec^−1^ and a negligible hysteresis value of ≈20 mV (inset graph in Figure 3b), which reflects the formation of a clean interface between the pV3D3 inter‐dielectric and the MoS_2_ channel.

As shown in Figure S7 (Supporting Information), to evaluate the interface quality between the pIL/HfO_2_ dielectric stack and MoS_2_, capacitance‐voltage (CV) measurements were performed on the four different metal–insulator–semiconductor (MIS) capacitors over a frequency range of 500 Hz to 500 kHz under varying *V*_TG_. The measured capacitances varied smoothly across the frequency range without any humps, even at the low‐frequency condition of 500 Hz, indicating excellent interface quality with minimal interface trap density between MoS_2_ and the pIL/HfO_2_ stack.^[^ ^33^ ^]^

In Figure 3c, the interface trap density (*D*_it_) was extracted as a function of energy using the high frequency and low frequency CV method (Hf–Lf method), with a detailed calculation procedure provided in Figure S7 (Supporting Information).^[^ ^34^ ^]^ In the depletion region of the MoS_2_ band (*E*_C_ – *E*_T_ ≈ 0.4 eV), the average *D*_it_ (*D*_it,avg_) was extracted to be 8.9 × 10^10^ cm^−2^ e^−1^ V^−1^, which is in good agreement with the *D*_it_ value (10^11^ cm^−2^ e^−1^ V^−1^) estimated from the *SS*_min_ (60.9 mV dec^−1^) of the transfer curve in Figure 3b based on Equation 1 below.^[^ ^35^ ^]^

(1) $$C_{it}=qD_{it}=C_{ox}\left(\frac{SS}{\ln\left(10\right)}\cdot \frac{q}{kT}-1\right)$$

This low *D*_it_ characteristic is highly competitive compared to previously reported top‐gate MoS_2_ transistors, as summarized in Table S1 (Supporting Information). Such improvement is attributed to the non‐polar nature of the pIL, which suppresses the formation of hydroxyl‐related traps, and the surface‐independent conformality of the iCVD process,^[^ ^16^, ^17^ ^]^ both of which contribute to reducing interfacial potential fluctuations induced by dielectric defects and thereby effectively suppress the formation of long band‐tail states within the MoS_2_ band.^[^ ^36^ ^]^

Utilizing our double‐gate structured MoS_2_ transistor, the electrical property of the MoS_2_ top‐gate transistor could be controlled through applying various back‐gate voltage biases (*V*_BG_).^[^ ^13^, ^37^ ^]^ Figure 3d shows the transfer characteristic of the underlap‐TG MoS_2_ device as a function of *V*_TG_ under *V*_BG_ ranging from 0 to 20 V at a step of 5 V with fixed drain to source votlage (*V*_DS_) of 1 V. As *V*_BG_ increases, the carrier density in MoS_2_ is enhanced, resulting in the increase of I_ON_ with the linear negative shift of top‐gate threshold voltage (*V*_TH, TG_).

In Figure 3e, we investigated the variation of the *V*_TH, TG,_ and the average *SS* (*SS*_avg_) corresponding to a four‐order increase in I_DS_ with respect to various applied *V*_BG_ (*V*_BG_: 0 to 20 V). As *V*_BG_ increases, *V*_TH, TG_ exhibits a linear negative shift trend without any notable degradation in switching properties, with *SS*_avg_ maintained below 90 mV dec^−1^ under all *V*_BG_ conditions. The slope (red dot line) observed in Figure 3e represents the coupling ratio between the capacitance of the back‐gate insulator (*C*_BG_) and the top‐gate insulator stack (*C*_TG_),^[^ ^38^ ^]^ which is calculated to be 0.052 (*C*_BG_/*C*_TG_). Considering the thickness and dielectric permittivity of the SiO_2_ back‐gate insulator as 90 nm and 3.9, respectively, the *C*_BG_/*C*_TG_ ratio allows us to estimate *C*_TG_ as 0.74 µF cm^−2^, which corresponds to EOT of ≈4.65 nm. Figure 3f shows the corresponding output characteristics of the top‐gate transistor as a function of *V*_DS_ for various *V*_TG_ ranging from 0 to 1.5 V in 0.25 V increments with an applied *V*_BG_ of 10 V, demonstrating a stable current modulation and saturation behavior of the double gate device.

In conventional MoS_2_ top‐gate transistor structures, low on‐current performance—primarily due to series resistance in the access region and contact resistance—remains a critical bottleneck, limiting the reliable operation of MoS_2_‐based electronic devices.^[^ ^8^, ^18^ ^]^ To address this limitation and verify the feasibility of basic logic circuit operation based on MoS_2_ top‐gate transistors incorporating the pIL insertion technique, we adopted a source/drain‐first strategy to fabricate a top‐gate MoS_2_ transistor where the gate overlaps the source/drain electrodes (overlap‐TG) (Figure S6b, Supporting Information). This design helps reduce access resistance through electrostatic doping induced by the gate electric field^[^ ^39^ ^]^ and lowers the Schottky barrier height (SBH, Φ_SB_) between the channel and source/drain electrodes by leveraging the van der Waals contact between Au and MoS_2_.^[^ ^40^ ^]^

**Figure 4:**
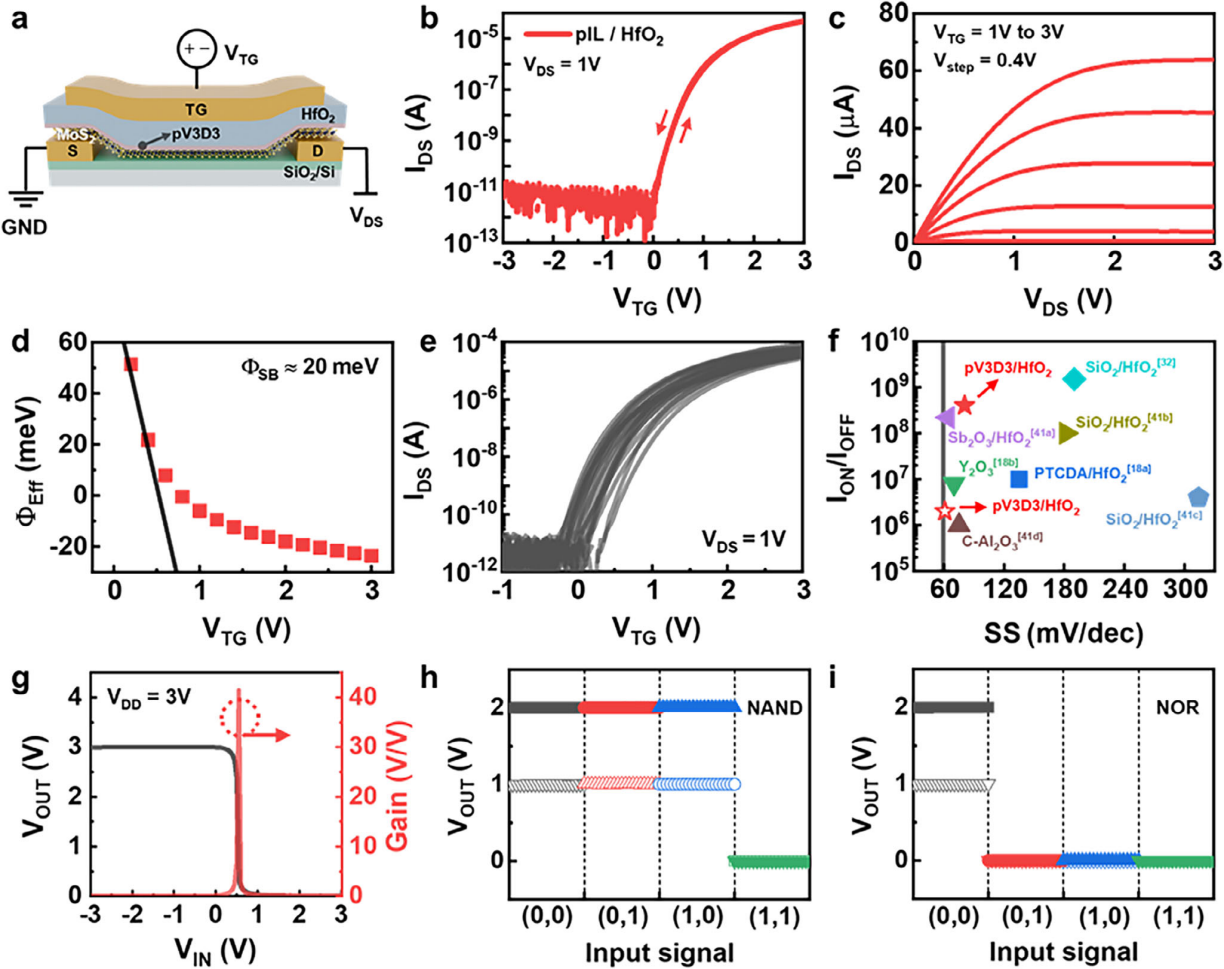
a) Schematic illustration of the overlap‐TG MoS_2_ transistor with a pIL/HfO_2_ dielectric stack. b) Dual sweep transfer characteristics of overlap‐TG MoS_2_ transistor with pIL/HfO_2_ gate insulators at *V*_DS_ of 1 V. (Channel length/width: 15/40 µm) c) Output characteristics of the overlap‐TG MoS_2_ transistor with the pIL/HfO_2_ gate stack. d) Schottky barrier height (SBH) as a function of *V*_TG_ calculated from Arrhenius plots in Figure S13 (Supplementary Information). e) Transfer characteristics of 23 overlap‐TG MoS_2_ transistors in a single batch. f) Comparison of *I*_ON_/*I*_OFF_ ratio and *SS* values between our MoS_2_ top‐gate transistors (represented by open and closed star symbols for overlap‐TG and underlap‐TG, respectively) and state‐of‐the‐art CVD‐grown MoS_2_ top‐gate transistors.^[^ ^18^, ^38^, ^48^ ^]^ g) The voltage transfer characteristics and voltage gain of an inverter as a function of *V*_IN_ at *V*_DD_ = 3 V. Output characteristics for h) NAND and i) NOR gates corresponding to various input signals ((0,0), (0,1), (1,0), and (1,1)) with open and closed symbols representing operation at *V*_DD_ = 1 and 2 V, respectively.

**Figure** **4**a illustrates the schematic of the fabricated MoS_2_ device with the overlap‐TG structure. The process begins with Cr/Au deposition on a SiO_2_ substrate, followed by MoS_2_ transfer to form a van der Waals contact between the source/drain metal and MoS_2_ channel. A pIL/HfO_2_ gate dielectric stack is subsequently formed using iCVD/ALD, and finally, a Cr/Au gate electrode is defined to complete the transistor. Detailed fabrication steps are provided in the Experimental Section.

To investigate the atomic‐scale quality of the pIL/HfO_2_ dielectric stack on MoS_2_, the cross‐sectional interface of the stacked layers was characterized using scanning transmission electron microscopy with energy‐dispersive X‐ray spectroscopy mapping in Figure S8 (Supporting Information). The 2.4 nm of pIL and 6 nm of HfO_2_ layers stacked on the MoS_2_ surface are clearly identified, indicating uniform coverage of the dielectric stack across the entire MoS_2_ surface without damaging the underlying MoS_2_ channel.

Figure 4b and Figure S9 (Supporting Information) show the transfer characteristics of the overlap‐TG MoS_2_ device with and without the pIL, respectively. In the absence of the pIL (Figure S9, Supporting Information), direct HfO_2_ deposition resulted in poor device performances, with *SS* of ≈458 mV dec^−1^, a large hysteresis value ≈200 mV, and high off‐state current above 100 pA. These limitations, which lead to high off‐state power consumption, are attributed to poor interface quality due to the incompatibility of ALD processes with 2D MoS_2_, along with uncontrollable charge transfer doping effect induced from HfO_2_.^[^ ^41^ ^]^

In contrast, as shown in Figure 4b, the insertion of the pIL significantly enhanced the device performance, yielding a *V*_TH_ of 0.9 V with normally‐off operation, a hysteresis of 32 mV, and a *SS*_min_ of 80.6 mV dec^−1^ (Figure S10, Supporting Information). Those excellent electrical characteristics of the pIL‐inserted overlap‐TG MoS_2_ transistor were further confirmed by measurements using a high‐resolution source measurement unit, as presented in Figure S11 (Supporting Information), where consistent electrical performances were observed across 13 different devices. Furthermore, the pIL/HfO_2_ top‐gate insulator stack exhibited highly robust insulating behavior, maintaining a gate leakage current (*I*_TG_) density below 2 µA/cm^2^ across the entire *V*_TG_ range when normalized to the channel area. (Figure S11b, Supporting Information)

In Figure S12 (Supporting Information), low‐frequency noise (LFN) measurements were conducted to further investigate the charge transport behavior of the overlap‐TG MoS_2_ transistor incorporating the pIL. The LFN analysis revealed a typical 1/*f* ^γ^ dependency. (γ ≈ 0.9–1) The normalized noise power spectral density (*S*_ID_/*I*_D_^2^) exhibited a clear g_m_/I_D_ dependence, consistent with the Hooge mobility fluctuation model where the Hooge parameter was calculated to be 0.036, which is comparable with previously reported research.^[^ ^13^, ^42^ ^]^ These results suggest that the contribution of carrier number fluctuations due to trapping is relatively minimal, and the dominant noise source arises from mobility fluctuations induced by carrier scattering near the channel.^[^ ^43^ ^]^

These results confirm the excellent interface quality between the pIL and MoS_2_, resulting from the superior compatibility of the iCVD process with 2D MoS_2_ compared to the ALD process. Furthermore, as discussed in Figure 2, the pIL serves as an effective tunneling barrier that suppresses charge transfer doping from HfO_2_, thereby enabling a positive *V*_TH_ and normally‐off operation, with the turn‐on voltage located near 0 V, highlighting its strong potential for low‐power TFT applications.^[^ ^44^ ^]^

Notably, the overlap‐TG structured MoS_2_ transistor exhibited markedly enhanced electrical performance in terms of *I*_ON_ and µ_FE_ compared to the underlap‐TG MoS_2_ transistor, achieving an *I*_ON_/*I*_OFF_ ratio exceeding 10^8^ and a µ_FE_ of 19.2 cm^2 ^V^−1^ s^−1^, while maintaining stable output characteristics with reliable current modulation, as presented in Figure 4c. These improvements are attributed to reduced series resistance and lowered SBH, enabled by the overlap‐TG structure. Figure 4d presents the effective Schottky barrier height (Φ_Eff_) of the overlap‐TG device as a function of *V*_TG_, extracted from the Arrhenius plots shown in Figure S13 (Supporting Information). The results reveal an SBH of 20 meV between Au and MoS_2_, which is markedly lower than the value predicted by the Schottky‐Mott rule.^[^ ^45^ ^]^ This low SBH is attributed to the formation of a van der Waals contact between polycrystalline MoS_2_ and Au, which effectively suppresses metal‐induced gap states and transitions the dominant interface mechanism to defect‐induced gap states.^[^ ^46^ ^]^ As a result, the Au work function aligns with shallow states within the MoS_2_ band, leading to a reduced SBH at the Au/MoS_2_ contact interface.^[^ ^40^ ^]^

Also, as shown in Figure S14 (Supporting Information), further enhancement in on‐current was observed by adopting a shorter channel length (3 µm) in the source/drain‐first structure. However, a noticeable reduction in the µ_FE_ was observed (Figure S14b, Supporting Information), which is likely attributed to the increasing influence of contact resistance as the contribution of the intrinsic channel resistance diminished.^[^ ^47^ ^]^ These results indicate that, for short‐channel top‐gate MoS_2_ transistors, achieving higher drive current requires not only dielectric and structural optimization but also systematic engineering of the contact resistance at the channel–metal interface.

To evaluate the scalability of our pIL insertion technique for demonstrating top‐gate MoS_2_ transistors, 30 devices were fabricated in a single batch, and the transfer characteristics of 23 devices that exhibited proper functionalities were depicted in Figure 4e. All devices operate in enhancement mode, exhibiting a narrow *V*_TH_ distribution with an average *V*_TH_ value of 0.9 V, a high *I*_ON_/*I*_OFF_ value above 10^7,^ and an average µ_FE_of 16 cm^2^ V^−1^ s^−1^ (Figure S15, Supporting Information). The demonstrated scalability of the iCVD‐pIL insertion technique in MoS_2_ top‐gate transistors highlights its strong potential for enabling large‐area, low‐power integrated MoS_2_ TFT circuits, especially when combined with wafer‐scale MoS_2_ synthesis technologies.

Furthermore, the benchmark graph presented in Figure 4f confirms that our devices deliver a highly competitive *I*_ON_/*I*_OFF_ ratio and *SS* compared to other state‐of‐the‐art CVD‐grown MoS_2_ top‐gate transistors.^[^ ^18^, ^38^, ^48^ ^]^ These results validate the technological promise of the pIL engineering strategy for scalable MoS_2_ top‐gate transistor integration, enabling low‐power operation.

To further verify the scalability and practical applicability of the pIL insertion technique, we successfully demonstrated fundamental logic gate circuits, including NOT (inverter), NAND, and NOR gates, using the overlap‐TG MoS_2_ transistors. Figure S16 (Supporting Information) shows an OM image and a schematic illustration of the fabricated logic gate circuits based on our overlap‐TG MoS_2_ transistors. Figure 4g illustrates the voltage transfer characteristics of an NMOS inverter logic circuit, constructed using two overlap‐TG MoS_2_ transistors. As shown in Figure S16a (Supporting Information), in our fabricated inverter circuit, one transistor functions as a load TFT, operating as a pull‐up transistor by connecting the gate and source electrode, while the other transistor serves as the driving TFT to control the output signals of the inverter. Under varying supply voltages (*V*_DD_) of 3 V, the inverter demonstrates its ability to transition from a logic “0” (low *V*_OUT_) to a logic “1” (high *V*_OUT_) in response to input voltages (*V*_IN_) with a maximum output gain of 41 V/V. Figure S17 (Supporting Information) shows the voltage transfer characteristics of the inverter and corresponding output voltage gain for various *V*_DD_ ranging from 1 to 3 V, demonstrating that the inverter could stably function in operating voltage as low as 1 V.

Figure 4h,i demonstrates the NAND and NOR logic gate circuits implemented using the overlap‐TG MoS_2_ transistors. The NAND logic gate utilizes a series configuration of transistors in the pull‐down network, while the NOR logic gate employs a parallel configuration as schematically illustrated in Figure S16b,c (Supporting Information), respectively. By implementing logic “1” and “0” with *V*_IN_ of ±1 V at *V*_DD_ = 1 V and *V*_IN_ of ±2 V at *V*_DD_ = 2 V, both NAND and NOR gates demonstrated correct functionality, delivering output signals aligned with their expected truth tables.

Our demonstrated device, incorporating a 2.4 nm pIL, exhibits a normally‐off characteristic, excellent switching behavior, and high µ_FE_, which are advantageous for low‐power operation. In Figure S18 (Supporting Information), to further assess the effectiveness of the pIL in the overlap top‐gate MoS_2_ configuration, we systematically investigated the device performance as a function of reduced pIL thickness. As shown in Figure S18 (Supporting Information), we compared the electrical performance of MoS_2_ top‐gate transistors incorporating sub‐2 nm pIL interlayers. As the pIL thickness decreased, the suppression of electron tunneling from the HfO_2_ layer became less effective, resulting in a negative shift in *V*_TH,_ and when the pIL thickness was reduced below 1 nm, device degradation began to emerge. Nevertheless, even with a pIL thickness of ≈1 nm, the top‐gate MoS_2_ transistor exhibited excellent performance, achieving *SS*_min_ of 88.4 mV dec^−1^, negligible hysteresis (15 mV) at *V*_BG_ = 0 V, and a *V*_TH_ of 0.4 V, thereby enabling enhancement‐mode operation. Under these conditions, the reduced physical thickness of the top‐gate insulator allowed the EOT to be scaled down to 3.4 nm.

### 2.4 Demonstration of Low‐Power Flexible Top‐Gate MoS_2_ Transistor

Here, we demonstrate flexible MoS_2_ top‐gate transistors incorporating pIL and their integration into a flexible inverter circuit fabricated on a flexible polyimide (PI) substrate, enabling a comprehensive investigation of their mechanical flexibility and corresponding electrical performance. The detailed fabrication process is provided in the Experimental Section.

**Figure 5:**
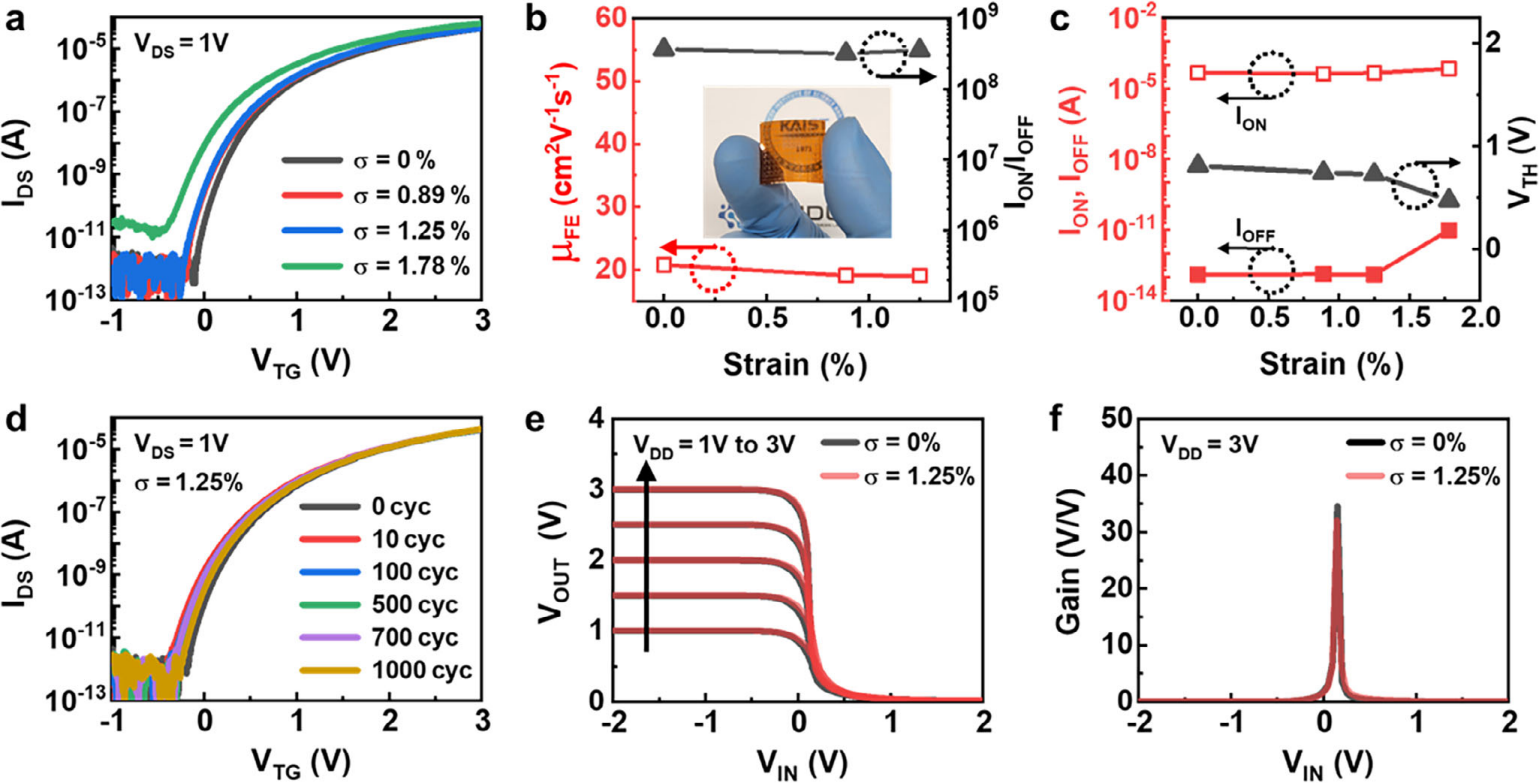
a) Transfer characteristics of the flexible MoS_2_ top‐gate transistor compared under various tensile strain conditions. Variations in b) *I*_ON_/*I*_OFF_ and µ_FE_, and c) *I*_ON_, *I*_OFF,_ and *V*_TH_ as a function of tensile strains ranging from 0% to 1.78%. d) Transfer characteristics of the flexible MoS_2_ top‐gate transistor estimated during 10^3^ bending cycles under a tensile strain of 1.25%. e) Voltage output characteristics of the flexible inverter as a function of *V*_IN_ at various *V*_DD_ ranging from 1 to 3 V with 0.5 V increment under 0% (black line) and 1.25% (red line) tensile strain conditions. f) Voltage gain characteristics as a function of *V*_IN_ at *V*_DD_ = 3 V, compared under 0% and 1.25% tensile strain conditions.

To assess the mechanical robustness of the flexible MoS_2_ transistors, **Figure** **5**a presents the transfer characteristics under varying tensile strain (ɛ) conditions ranging from 0% to 1.78%. The applied strain was calculated using the equation ɛ = t/2R, where t is the thickness of the PI substrate and R is the bending radius.^[^ ^49^ ^]^

As illustrated in Figure 5a, under flat conditions (ɛ = 0%), the device exhibited enhancement‐mode operation with *V*_TH_ of 0.8 V, *I*_ON_/*I*_OFF_ ratio of 2 × 10^8^, a *SS*_min_ of 66.7 mV dec^−1^, and µ_FE_ of 20.8 cm^2^ V^−1 ^s^−1^, while maintaining stable transfer characteristics without significant degradation under applied tensile strain conditions. Statistically, variations in µ_FE_, *I*_ON_/*I*_OFF_ ratio, and *V*_TH_ remained within ≈10% for tensile strains up to 1.25% (Figure 5b,c).

At a higher tensile strain of 1.78%, an increase in off‐state current (green line, Figure 5a) resulted in degradation of *I*_OFF_ (Figure 5c). This behavior is attributed to compromised insulating properties of the pIL/HfO_2_ dielectric stack under substantial strain, as further discussed in Figure S19 (Supporting Information). Nevertheless, mechanical durability can be potentially enhanced through neutral‐plane engineering, improving device suitability for flexible electronic applications.^[^ ^50^ ^]^

The device exhibited excellent bending endurance, maintaining consistent transfer characteristics without significant degradation after 10^3^ bending cycles under a tensile strain of 1.25% (Figure 5d). As depicted in Figure S20 (Supporting Information), both *I*_ON_/*I*_OFF_ ratio and µ_FE_ values remained within ≈10% of their initial values after cyclic bending tests, verifying the mechanical reliability and robustness of the demonstrated flexible MoS_2_ top‐gate transistor.

Furthermore, we successfully implemented a flexible inverter logic circuit based on the fabricated top‐gate MoS_2_ transistors. Figure 5e displays the voltage transfer characteristics of the inverter on a PI substrate, demonstrating stable logic states (“0” and “1”) over *V*_DD_ ranging from 1 to 3 V in both flat and 1.25% tensile‐strained conditions, without noticeable performance degradation.

Figure 5f illustrates the voltage gain of the inverter at *V*_DD_ = 3 V, achieving a maximum gain of 34.6 V/V under flat conditions and 32 V/V under 1.25% tensile strain, indicating minimal degradation (less than 10%) during bending. Additionally, Figure S21 (Supporting Information) confirms consistent voltage gain across all *V*_DD_ ranging from 1 to 3 V under a 1.25% tensile strain condition, validating the mechanical and operational reliability of the flexible inverter circuit.

To the best of our knowledge, our flexible MoS_2_ top‐gate transistor exhibits superior *SS*_min_ and *I*_ON_/*I*_OFF_ ratio, along with competitive µ_FE_ compared to the previously reported flexible MoS_2_ top‐gate transistors, as summarized in **Table** **1**. Notably, our device achieves a low off‐state current of 6.5 × 10^−7^ µA µm^−1^ at *V*_TG_ = 0 V, validating its normally‐off characteristic, which is a key requirement for enabling low‐power flexible electronic systems.

**Table 1:** Comparison of our top‐gate MoS_2_ flexible transistor with other previously reported devices.

Channel	Dielectric	*I*_ON_/*I*_OFF_	*SS*_min_ [mV/dec]	µ_FE_ [cm^2^ V^−1^ s^−1^]	*I*_OFF_ [A/µm] @ *V*_TG_ = 0V	Gain [V/V] [*V*_DD_]	Refs.
CVD MoS_2_	pV3D3/ HfO_2_	2 × 10^8^	66.7	20.8	5 × 10^−13^	34.6 (3 V)	This work
CVD MoS_2_	Ion Gating	10^3^	–	3.01	–	–	[51]
CVD MoS_2_	Al_2_O_3_	10^6^	–	9	10^−9^	9 (5 V)	[52]
CVD MoS_2_	Al_2_O_3_	10^8^	370	9.1	6 × 10^−14^	7 (5 V)	[9a]
CVD MoS_2_	Al_2_O_3_	10^5^	850	24	3 × 10^−6^	–	[53]
Exfoliated MoS_2_	Al_2_O_3_	10^7^	158	–	2 × 10^−8^	9 (5 V)	[54]
Exfoliated MoS_2_	Al_2_O_3_	10^6^	250	19	10^−7^	–	[55]
CVD MoS_2_	HfO_2_	10^5^	–	–	10^−5^	–	[56]
CVD MoS_2_	Al_2_O_3_	10^7^	–	18	3 × 10^−11^	–	[57]

These outstanding device characteristics result from the excellent interfacial compatibility between the iCVD‐based pIL and the MoS_2_ channel, underscoring the effectiveness of pIL engineering for realizing high‐performance, low‐power, and mechanically robust MoS_2_ top‐gate transistors for next‐generation flexible integrated circuits.

## 3 Conclusion

In this study, we successfully integrated an iCVD‐based pV3D3 inter‐dielectric layer into MoS_2_ top‐gate transistors, effectively overcoming key challenges in dielectric compatibility and interface engineering for 2D MoS_2_. The pV3D3 layer functioned as an effective buffer, enabling conformal ALD‐HfO_2_ deposition on MoS_2_ while suppressing charge transfer doping and interfacial defect formation induced by HfO_2_.

Top‐gate MoS_2_ transistors with an underlap structure incorporating the pV3D3 layer demonstrated excellent performance, including a high *I*_ON_/*I*_OFF_ ratio (>10^6^), near‐ideal *SS*_min_ of 60.9 mV dec^−1^, and a low interface trap density (*D*_it,avg_ ≈ 8.9 × 10^10^ cm^−2^ e^−1^ V^−1^). Further improvements were achieved with the overlap‐TG structure, which reduced series resistance and Schottky barrier height, yielding an *I*_ON_/*I*_OFF_ ratio exceeding 10^8^, a high µ_FE_of 19.2 cm^2 ^V^−1 ^s^−1^ with normally‐off operation mode.

The scalability of the proposed dielectric strategy was validated through the successful implementation of logic circuits—including inverters, NAND, and NOR gates—exhibiting stable and reproducible operation. Additionally, flexible top‐gate MoS_2_ transistors fabricated on polyimide substrates retained their electrical performance under tensile strains up to 1.25% and after 10^3^ bending cycles, highlighting the effectiveness of the pV3D3 inter‐dielectric for realizing high‐performance MoS_2_‐based flexible electronics.

These results establish iCVD‐based pV3D3 as a promising and scalable inter‐dielectric solution for realizing high‐performance and low‐power top‐gate MoS_2_ transistors, paving the way for the development of next‐generation functional electronic devices.

## 4 Experimental Section

### MoS_2_ Synthesis

The monolayer MoS_2_ film was synthesized within the inner quartz tube (2 inches) situated in the outer quartz tube (4 inches) furnace. High‐purity argon gas, serving as a carrier gas, flowed through the 4‐inch quartz tube at a pressure of 750 Torr. 120 mg of sulfur powder was positioned upstream of the furnace (Heater 1). In the downstream section of the furnace (Heater 2), 3.0–3.5 mg of molybdenum trioxide (MoO_3_) powder was placed, while the Si/SiO_2_ growth substrate was oriented facing downward. Note that perylene 3,4,9,10‐tetracarboxylic acid tetrapotassium salt (PTAS) was applied as a seeding promoter on the growth substrate. Subsequently, Heater 1 and Heater 2 were raised to temperatures of 830 and 320 °C, respectively, during the primary phase of MoS_2_ growth.

### Device Fabrication and Characterization

To prepare the Si/SiO_2_ substrate, it was cleaned using a piranha solution (H_2_SO_4_:H_2_O_2_ = 1:1) for 20 min, followed by thorough rinsing with deionized water (DI). In the overlap‐TG configuration, the source and drain electrodes, comprising Cr (5 nm)/Au (35 nm), were deposited first using a thermal evaporator. Conversely, for the underlap‐TG configuration, the source and drain electrodes were deposited after the MoS_2_ channel formation, as described in the subsequent section, with Cr (15 nm)/Au (35 nm) deposition. For the overlap‐TG configuration, MoS_2_ synthesized via chemical vapor deposition (CVD) was transferred onto the source and drain electrodes on the prepared substrate using a polystyrene(PS)‐assisted transfer method, with the pattern defined by GXR601 photoresist. In the underlap‐TG configuration, MoS_2_ was initially transferred onto the prepared SiO_2_ substrate. After transferring the MoS_2_ channel, the channel region was defined using an ICP asher. Following this, pV3D3 was deposited as an interlayer dielectric via initiated chemical vapor deposition (iCVD). During this process, 300 sccm of the monomer (V3D3) and 100 sccm of the initiator (TBPO) were simultaneously introduced, while maintaining a substrate temperature of 40 °C and a filament temperature of 250 °C. Subsequently, HfO_2_ was deposited on top of the pV3D3 using atomic layer deposition (ALD) at 150 °C. Finally, the gate electrode, consisting of Cr (15 nm)/Au (50 nm), was patterned on the fabricated substrate using photolithography with NR9 photoresist.

A flexible MoS_2_ top‐gate transistor was fabricated on a prepared 125 µm thick flexible PI substrate. The substrate was sequentially cleaned with acetone and isopropyl alcohol (IPA) using an ultrasonicator. After the cleaning process, a 2 µm thick planarization layer was formed by spin‐coating an epoxy photoresist (Microchem Corporation, SU‐8 2) onto the substrate, followed by UV exposure for cross‐linking. Cr (15 nm) /Au (35 nm) source and drain electrodes were subsequently deposited using thermal evaporation, and CVD‐grown MoS_2_ was transferred onto the electrodes to define the channel region. The top‐gate dielectric stack, comprising a 2 nm pV3D3 layer deposited via iCVD and a 6 nm HfO_2_ layer deposited via ALD, was formed. Finally, Cr (15 nm) /Au (50 nm) gate electrodes were deposited to complete the device structure.

A parameter analyzer (Keithley 4200 SCS) and probe station (MS‐TECH, MST‐1000B) were used for standard electrical measurements, while a high‐resolution source measurement unit (Keysight B1500A) was employed for precision characterization. Note that all measurements were implemented in the dark and a vacuum condition (≈5 × 10^−2^ Torr).

In the in‐house micro‐PL and Raman experiments, scattered spectra were collected using a spectrometer (Andor, Kymera 328i) coupled with a charge‐coupled device (Andor iDus DU401ABVF). The excitation laser wavelength for both photoluminescence (PL) and Raman spectroscopy was set at 532 nm. Each measurement for PL and Raman had the same exposure time to ensure accurate intensity comparisons.

## Conflict of Interest

The authors declare no conflict of interest.

## Supporting information



Supporting Information

## Data Availability

The data that support the findings of this study are available from the corresponding author upon reasonable request.

## References

[1] a) K. Myny , Nat. Electron. 2018, 1, 30;

[2] M. Halik , H. Klauk , U. Zschieschang , G. Schmid , C. Dehm , M. Schütz , S. Maisch , F. Effenberger , M. Brunnbauer , F. Stellacci , Nature 2004, 431, 963. 15496917 10.1038/nature02987

[3] a) S. Wang , J. Xu , W. Wang , G.‐J. N. Wang , R. Rastak , F. Molina‐Lopez , J. W. Chung , S. Niu , V. R. Feig , J. Lopez , Nature 2018, 555, 83; 29466334 10.1038/nature25494

[4] N. Li , Q. Wang , C. Shen , Z. Wei , H. Yu ,

[5] M. Chhowalla , D. Jena , H. Zhang , Nat. Rev. Mater. 2016, 1, 16052.

[6] a) S. Bertolazzi , J. Brivio , A. Kis , ACS Nano 2011, 5, 9703; 22087740 10.1021/nn203879f

[7] P.‐C. Shen , C. Su , Y. Lin , A.‐S. Chou , C.‐C. Cheng , J.‐H. Park , M.‐H. Chiu , A.‐Y. Lu , H.‐L. Tang , M. M. Tavakoli , Nature 2021, 593, 211. 33981050 10.1038/s41586-021-03472-9

[8] a) Y. Wang , J. C. Kim , R. J. Wu , J. Martinez , X. Song , J. Yang , F. Zhao , A. Mkhoyan , H. Y. Jeong , M. Chhowalla , Nature 2019, 568, 70; 30918403 10.1038/s41586-019-1052-3

[9] a) A. T. Hoang , L. Hu , B. J. Kim , T. T. N. Van , K. D. Park , Y. Jeong , K. Lee , S. Ji , J. Hong , A. K. Katiyar , Nat. Nanotechnol. 2023, 18, 1439; 37500777 10.1038/s41565-023-01460-w

[10] a) X. Li , J. Yang , H. Sun , L. Huang , H. Li , J. Shi , Adv. Mater. 2023, 2305115; 10.1002/adma.202305115 37406665

[11] Y. Y. Illarionov , T. Knobloch , M. Jech , M. Lanza , D. Akinwande , M. I. Vexler , T. Mueller , M. C. Lemme , G. Fiori , F. Schwierz , Nat. Commun. 2020, 11, 3385. 32636377 10.1038/s41467-020-16640-8 PMC7341854

[12] a) P. R. Pudasaini , A. Oyedele , C. Zhang , M. G. Stanford , N. Cross , A. T. Wong , A. N. Hoffman , K. Xiao , G. Duscher , D. G. Mandrus , Nano Res. 2018, 11, 722;

[13] a) X. Zou , C. W. Huang , L. Wang , L. J. Yin , W. Li , J. Wang , B. Wu , Y. Liu , Q. Yao , C. Jiang , Adv. Mater. 2016, 28, 2062; 26762171 10.1002/adma.201505205

[14] a) T.‐E. Lee , Y.‐C. Su , B.‐J. Lin , Y.‐X. Chen , W.‐S. Yun , P.‐H. Ho , J.‐F. Wang , S.‐K. Su , C.‐F. Hsu , P.‐S. Mao , in IEEE Int. Electron Devices Meet.(IEDM), IEEE , San Francisco, CA, USA, 2022;

[15] a) H.‐Y. Lan , J. Appenzeller , Z. Chen , in IEEE Int. Electron Devices Meet.(IEDM), IEEE, San Francisco, CA, USA, 2022;

[16] a) H. Moon , H. Seong , W. C. Shin , W.‐T. Park , M. Kim , S. Lee , J. H. Bong , Y.‐Y. Noh , B. J. Cho , S. Yoo , Nat. Mater. 2015, 14, 628; 25751074 10.1038/nmat4237

[17] a) H. Park , D. S. Oh , W. Hong , J. Kang , G. B. Lee , G. H. Shin , Y. K. Choi , S. G. Im , S. Y. Choi , Adv. Mater. Interfaces 2021, 8, 2100599;

[18] a) W. Li , J. Zhou , S. Cai , Z. Yu , J. Zhang , N. Fang , T. Li , Y. Wu , T. Chen , X. Xie , Nat. Electron. 2019, 2, 563;

[19] a) W. E. Tenhaeff , K. K. Gleason , Adv. Funct. Mater. 2008, 18, 979;

[20] M. J. Kim , J. Jeong , T. I. Lee , J. Kim , Y. Tak , H. Park , S. G. Im , B. J. Cho , Macromol. Mater. Eng. 2021, 306, 2000608.

[21] a) X. Wang , X. Luo , W. Du , Y. Shen , X. Huang , Z. Yang , J. Zhao , Int. J. Extreme Manufact. 2024, 6, 055101;

[22] a) S. Kim , S.‐H. Lee , I. H. Jo , T. J. Park , J. H. Kim , Appl. Surf. Sci. 2024, 645, 158790;

[23] H. Li , Q. Zhang , C. C. R. Yap , B. K. Tay , T. H. T. Edwin , A. Olivier , D. Baillargeat , Adv. Funct. Mater. 2012, 22, 1385.

[24] a) R. Saito , Y. Tatsumi , S. Huang , X. Ling , M. Dresselhaus , J. Phys.: Condens. Matter 2016, 28, 353002; 27388703 10.1088/0953-8984/28/35/353002

[25] a) K. M. Price , S. Najmaei , C. E. Ekuma , R. A. Burke , M. Dubey , A. D. Franklin , ACS Appl. Nano Mater. 2019, 2, 4085;

[26] a) C. J. McClellan , E. Yalon , K. K. Smithe , S. V. Suryavanshi , E. Pop , ACS Nano 2021, 15, 1587; 33405894 10.1021/acsnano.0c09078

[27] a) W. Scopel , R. Miwa , T. Schmidt , P. Venezuela , J. Appl. Phys. 2015, 117, 194303;

[28] Y. Li , X. Li , H. Chen , J. Shi , Q. Shang , S. Zhang , X. Qiu , Z. Liu , Q. Zhang , H. Xu , ACS Appl. Mater. Interfaces 2017, 9, 27402. 28796477 10.1021/acsami.7b08893

[29] S. Mouri , Y. Miyauchi , K. Matsuda , Nano Lett. 2013, 13, 5944. 24215567 10.1021/nl403036h

[30] a) K. M. McCreary , A. T. Hanbicki , S. V. Sivaram , B. T. Jonker , APL Mater. 2018, 6, 111106;

[31] I. Lee , M. Kang , S. Park , C. Park , H. Lee , S. Bae , H. Lim , S. Kim , W. Hong , S. Y. Choi , Small 2024, 20, 2305143. 10.1002/smll.202305143 37670210

[32] R. Acharya , B. Peng , P. K. Chan , G. Schmitz , H. Klauk , ACS Appl. Mater. Interfaces 2019, 11, 27104. 31267732 10.1021/acsami.9b04361 PMC6750643

[33] P. Bolshakov , P. Zhao , A. Azcatl , P. K. Hurley , R. M. Wallace , C. D. Young , Microelectron. Eng. 2017, 178, 190.

[34] a) P. Zhao , A. Khosravi , A. Azcatl , P. Bolshakov , G. Mirabelli , E. Caruso , C. L. Hinkle , P. K. Hurley , R. M. Wallace , C. D. Young , 2D Mater. 2018, 5, 031002;

[35] S. Kim , A. Konar , W.‐S. Hwang , J. H. Lee , J. Lee , J. Yang , C. Jung , H. Kim , J.‐B. Yoo , J.‐Y. Choi , Nat. Commun. 2012, 3, 1011. 22910357 10.1038/ncomms2018

[36] S. Ghatak , A. N. Pal , A. Ghosh , ACS Nano 2011, 5, 7707. 21902203 10.1021/nn202852j

[37] a) M. Lee , C. Y. Park , D. K. Hwang , M. Kim , Y. T. Lee , npj 2D Mater. Appl. 2022, 6, 45;

[38] C. Sheng , X. Wang , X. Dong , Y. Hu , Y. Zhu , D. Wang , S. Gou , Q. Sun , Z. Zhang , J. Zhang , Adv. Funct. Mater. 2024, 34, 2400008.

[39] J. Bae , H. Ryu , D. Kim , C. S. Lee , M. Seol , K. E. Byun , S. Kim , S. Lee , Adv. Mater. 2024, 36, 2314164. 10.1002/adma.202314164 38608715

[40] J. Kwon , M. Seol , J. Yoo , H. Ryu , D.‐S. Ko , M.‐H. Lee , E. K. Lee , M. S. Yoo , G.‐H. Lee , H.‐J. Shin , Nat. Electron. 2024, 7, 356.

[41] X. Li , X. Xiong , T. Li , S. Li , Z. Zhang , Y. Wu , ACS Appl. Mater. Interfaces 2017, 9, 44602. 29199423 10.1021/acsami.7b14031

[42] a) V. K. Sangwan , H. N. Arnold , D. Jariwala , T. J. Marks , L. J. Lauhon , M. C. Hersam , Nano Lett. 2013, 13, 4351; 23944940 10.1021/nl402150r

[43] J. Na , M.‐K. Joo , M. Shin , J. Huh , J.‐S. Kim , M. Piao , J.‐E. Jin , H.‐K. Jang , H. J. Choi , J. H. Shim , Nanoscale 2014, 6, 433. 24212201 10.1039/c3nr04218a

[44] a) S. H. Wu , X. Jia , X. Li , C. C. Shuai , H. C. Lin , M. C. Lu , T. H. Wu , M. Y. Liu , J. Wu , D. Matsubayashi , presented at 2017 Symposium on VLSI Circuits IEEE, Kyoto, Japan, 2017;

[45] Y. Liu , J. Guo , E. Zhu , L. Liao , S.‐J. Lee , M. Ding , I. Shakir , V. Gambin , Y. Huang , X. Duan , Nature 2018, 557, 696. 29769729 10.1038/s41586-018-0129-8

[46] B.‐K. Kim , T.‐H. Kim , D.‐H. Choi , H. Kim , K. Watanabe , T. Taniguchi , H. Rho , J.‐J. Kim , Y.‐H. Kim , M.‐H. Bae , npj 2D Mater. Appl. 2021, 5, 9.

[47] H. Klauk , G. Schmid , W. Radlik , W. Weber , L. Zhou , C. D. Sheraw , J. A. Nichols , T. N. Jackson , Solid‐State Electron. 2003, 47, 297.

[48] a) Y. Xu , T. Liu , K. Liu , Y. Zhao , L. Liu , P. Li , A. Nie , L. Liu , J. Yu , X. Feng , Nat. Mater. 2023, 22, 1078; 37537352 10.1038/s41563-023-01626-w

[49] H. Park , D. S. Oh , K. J. Lee , D. Y. Jung , S. Lee , S. Yoo , S.‐Y. Choi , ACS Appl. Mater. Interfaces 2020, 12, 4749. 31896251 10.1021/acsami.9b18945

[50] a) Y.‐H. Kim , E. Lee , J. G. Um , M. Mativenga , J. Jang , Sci. Rep. 2016, 6, 25734; 27165715 10.1038/srep25734 PMC4863145

[51] J. Pu , Y. Yomogida , K.‐K. Liu , L.‐J. Li , Y. Iwasa , T. Takenobu , Nano Lett. 2012, 12, 4013. 22799885 10.1021/nl301335q

[52] S. M. Shinde , T. Das , A. T. Hoang , B. K. Sharma , X. Chen , J. H. Ahn , Adv. Funct. Mater. 2018, 28, 1706231.

[53] A. Daus , S. Vaziri , V. Chen , Ç. Köroğlu , R. W. Grady , C. S. Bailey , H. R. Lee , K. Schauble , K. Brenner , E. Pop , Nat. Electron. 2021, 4, 495.

[54] R. Cheng , S. Jiang , Y. Chen , Y. Liu , N. Weiss , H.‐C. Cheng , H. Wu , Y. Huang , X. Duan , Nat. Commun. 2014, 5, 5143. 25295573 10.1038/ncomms6143 PMC4249646

[55] G. A. Salvatore , N. Munzenrieder , C. Barraud , L. Petti , C. Zysset , L. Buthe , K. Ensslin , G. Troster , ACS Nano 2013, 7, 8809. 23991756 10.1021/nn403248y

[56] H.‐Y. Chang , M. N. Yogeesh , R. Ghosh , A. Rai , A. Sanne , S. Yang , N. Lu , S. K. Banerjee , D. Akinwande , Adv. Mater. 2016, 28, 1818. 26707841 10.1002/adma.201504309

[57] M. Choi , S.‐R. Bae , L. Hu , A. T. Hoang , S. Y. Kim , J.‐H. Ahn , Sci. Adv. 2020, 6, 5898. 10.1126/sciadv.abb5898 PMC7455500 32923597

