# Supplementary material for: Scalable Inter‐Dielectric Engineering via Vapor‐Phase Synthesis Process for Top‐Gate MoS2 Thin‐Film Transistor
Source: Small. 2025 Aug 26;21(42):e06282. doi: 10.1002/smll.202506282 (PMC12548015; doi:10.1002/smll.202506282)
Supplement: Supplementary file 1 — Supporting Information [file SMLL-21-e06282-s001.docx]

Supporting Information

Scalable Inter-Dielectric Engineering via Vapor-Phase Synthesis Process for Top-Gate MoS_2_ Thin-Film Transistor

Seohak Park^1^, Mingu Kang^1^, Inseong Lee^1^, Seungsun Yoo^2^, Sejin Kim^1^, Hyeongjin Lim^1^, Woonggi Hong^3^, Min Ju Kim^3^, Cheolmin Park^1^, Jeoungmin Ji^1^, Seunghyup Yoo^1^, and Sung-Yool Choi^1,2^*

S. Park, M. Kang, I. Lee, S. Kim, H. Lim, C. Park, J. Ji, S. Yoo and S.-Y. Choi

^1^School of Electrical Engineering, Korea Advanced Institute of Science and Technology (KAIST), 291 Daehak-ro, Yuseong-gu, Daejeon, 34141, Republic of Korea

Email: [sungyool.choi@kaist.ac.kr](mailto:sungyool.choi@kaist.ac.kr)

S. Yoo and S.-Y. Choi

^2^Graduate School of Semiconductor Technology, Korea Advanced Institute of Science and Technology (KAIST), 291 Daehak-ro, Yuseong-gu, Daejeon 34141, Republic of Korea

W. Hong and M. J. Kim

^3^Department of Electronics and Electrical Engineering, Department of Convergence Semiconductor Engineering, Dankook University, Yongin-si, Gyeonggi-do 16890, Republic of Korea

**Table S1. Performance comparison of top-gate MoS_2_ transistors.**

| **Channel** | **Dielectric** | **Method** | ***I*_ON_/*I*_OFF_** | ***SS*_min_**  **(mV/dec)** | ***D*_it_**  **(cm^-2^e^-1^V^-1^)** | **EOT**  **(nm)** | **Ref.** |
| --- | --- | --- | --- | --- | --- | --- | --- |
| CVD  MoS_2_ | pV3D3  / HfO_2_ | iCVD  /ALD | 2×10^6^ | 60.9 | Avg. *D*_it_  8.9×10^10^ | 4.65 | This work |
| CVD  MoS_2_ | PTCDA  / HfO_2_ | CVD  /ALD | 10^7^ | 135 | - | 2 | [1] |
| Exfoliated  MoS_2_ | PTCDA  / HfO_2_ | CVD  /ALD | 10^7^  (*V*_BG_ = 15V) | 60  (*V*_BG_ = 15V) | 8×10^11^ | 1 | [1] |
| CVD  MoS_2_ | Y_2_O_3_ | Transfer | >10^6^  (*V*_BG_ = 80V) | 70 | - | - | [2] |
| Exfoliated  MoS_2_ | Y_2_O_3_ | Transfer | 10^5^ | 60 | 5×10^11^ | 4.2 | [2] |
| CVD  MoS_2_ | Sb_2_O_3_  / HfO_2_ | Evaporation  /ALD | 2.2×10^8^ | 64 | - | 1.01 | [3] |
| Exfoliated  MoS_2_ | Sb_2_O_3_  / HfO_2_ | Evaporation  /ALD | >10^7^ | 60 | 1.4×10^11^ | 0.67 | [3] |
| CVD  MoS_2_ | Al_2_O_3_ | Transfer | 2×10^6^ | 68 | 7.6×10^9^ | ≈ 5 | [4] |
| CVD  MoS_2_ | SiO_2_  / HfO_2_ | Evaporation  /ALD | 1.5×10^9^ | 190 | - | - | [5] |
| Exfoliated  MoS_2_ | Er_2_O_3_ | Evaporation | 10^6^ | 90 | 6×10^11^ | 1.1 | [6] |
| CVD  MoS_2_ | AlO_x_  / HfO_2_ | Evaporation  /ALD | 10^8^ | 156 | - | - | [7] |
| CVD  MoS_2_ | SiO_2_  / HfO_2_ | Evaporation  /ALD | 4×10^6^ | 314 | - | 6.65 | [8] |
| CVD  MoS_2_ | C- Al_2_O_3_ | Transfer | >10^6^ | 75 | - | 1.4 | [9] |
| Exfoliated  MoS_2_ | C- Al_2_O_3_ | Transfer | 2.4×10^8^ | 61 | 8.4×10^9^ | 1.4 | [9] |

**
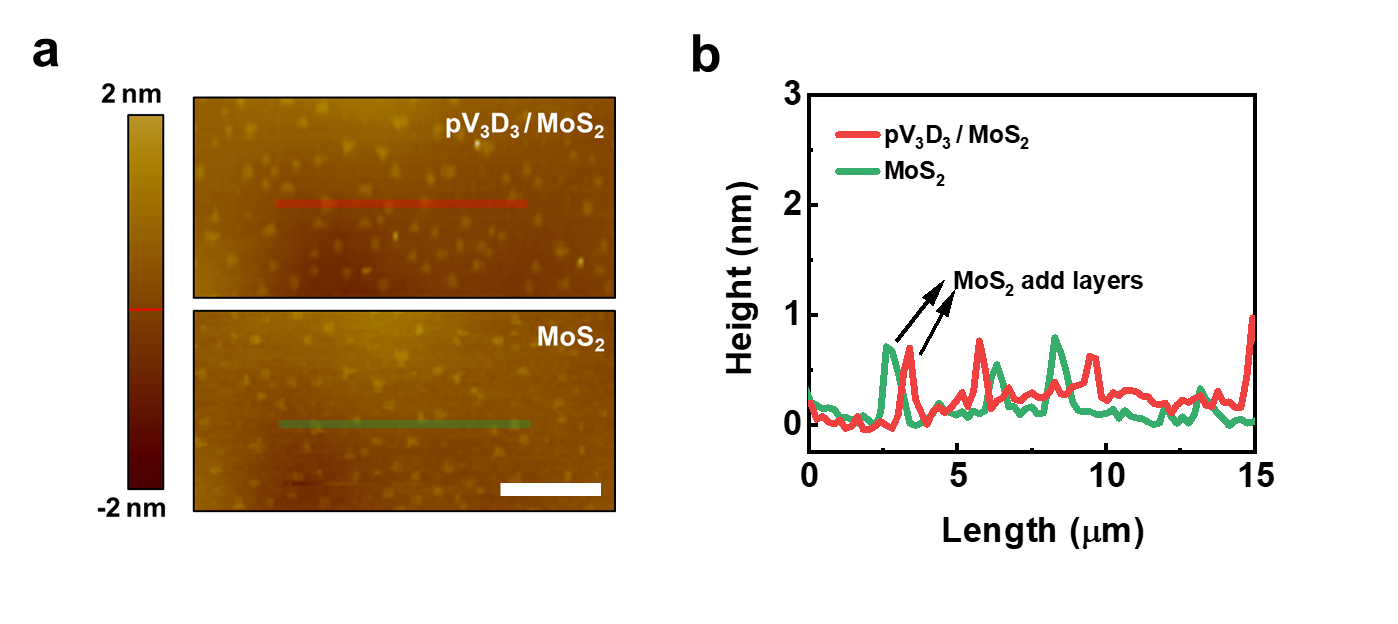
**

**Figure S1.** a) Atomic force microscope (AFM) images showing the surface of pristine MoS_2_ and the surface of MoS_2_ after the deposition of 2 nm pV3D3 (Scale bar: 10 μm). b) Height profiles of both samples derived from the line scan results indicated in the AFM images. (red line: pV3D3/MoS_2_, green line: MoS_2_)


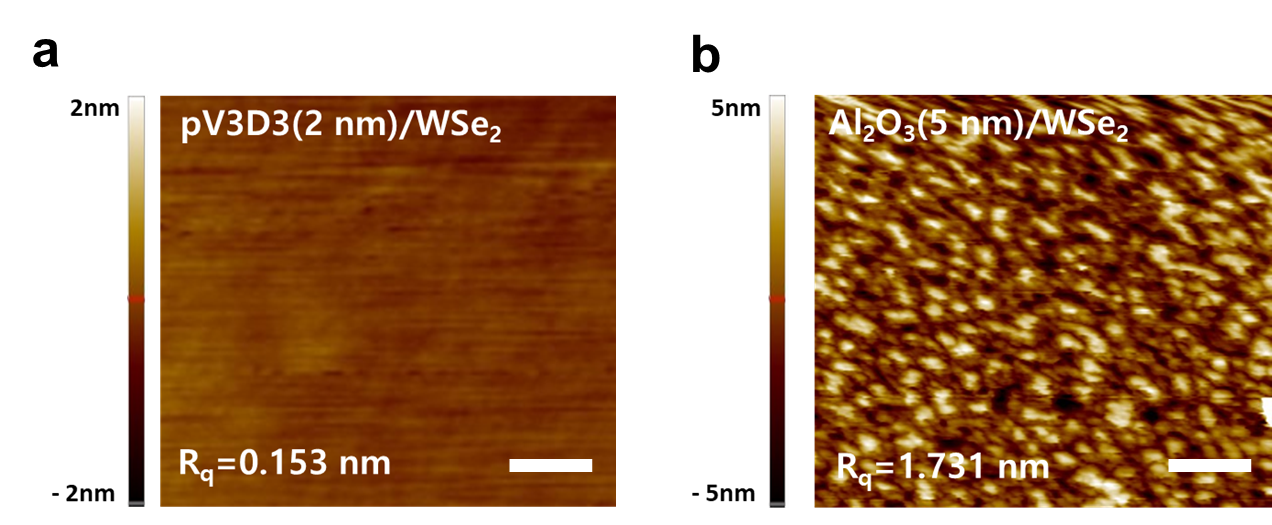


**Figure S2.** AFM images of exfoliated WSe_2_ with a) 2 nm pV3D3 (*R*_q_ ≈ 0.15 nm) by iCVD and b) 5 nm Al_2_O_3_ (*R*_q_ ≈ 1.73 nm) by ALD at 150 °C. (Scale bar: 2 μm)


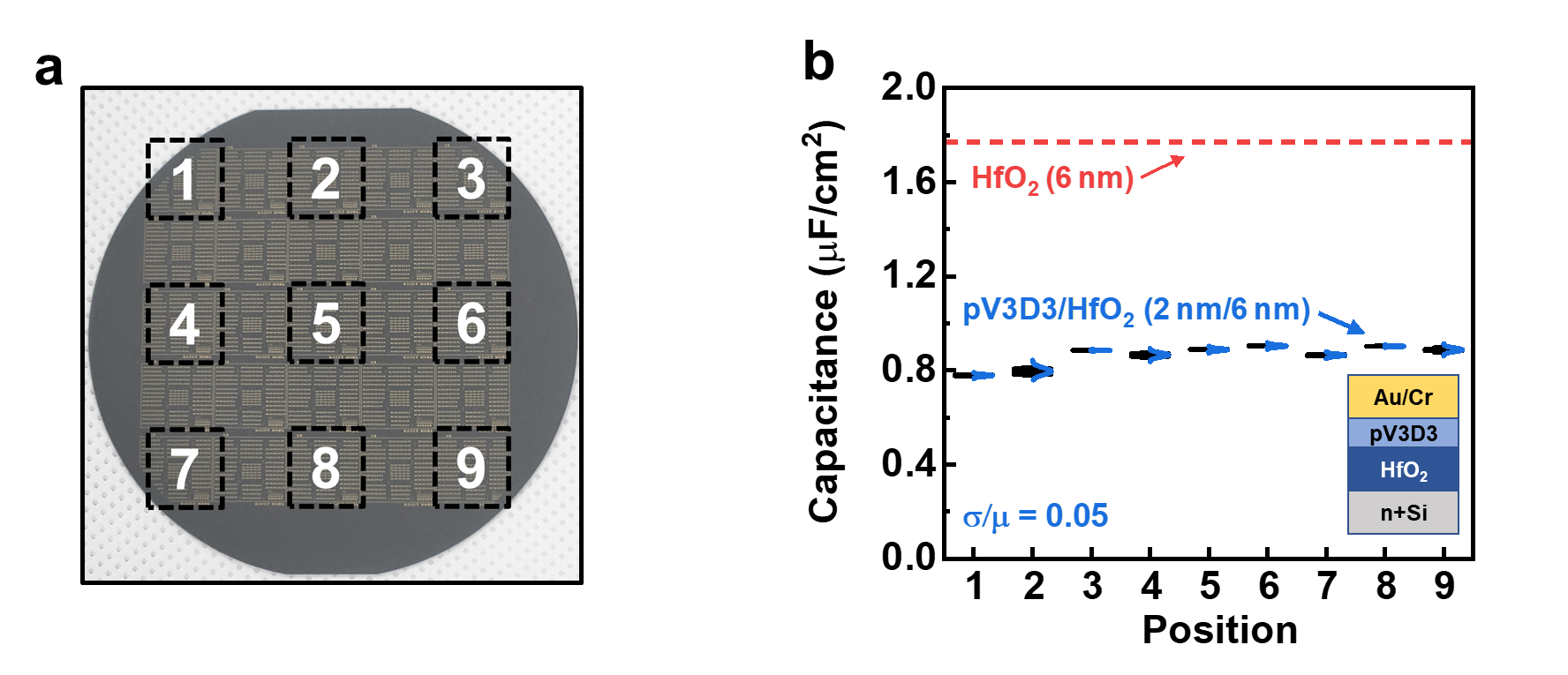


**Figure S3.** a) Optical image of the fabricated pV3D3 (2 nm)/HfO_2_ (6 nm) bilayer capacitors on a 4-inch wafer. b) Comparison of capacitance values from five randomly selected capacitors at each of nine different positions.

**
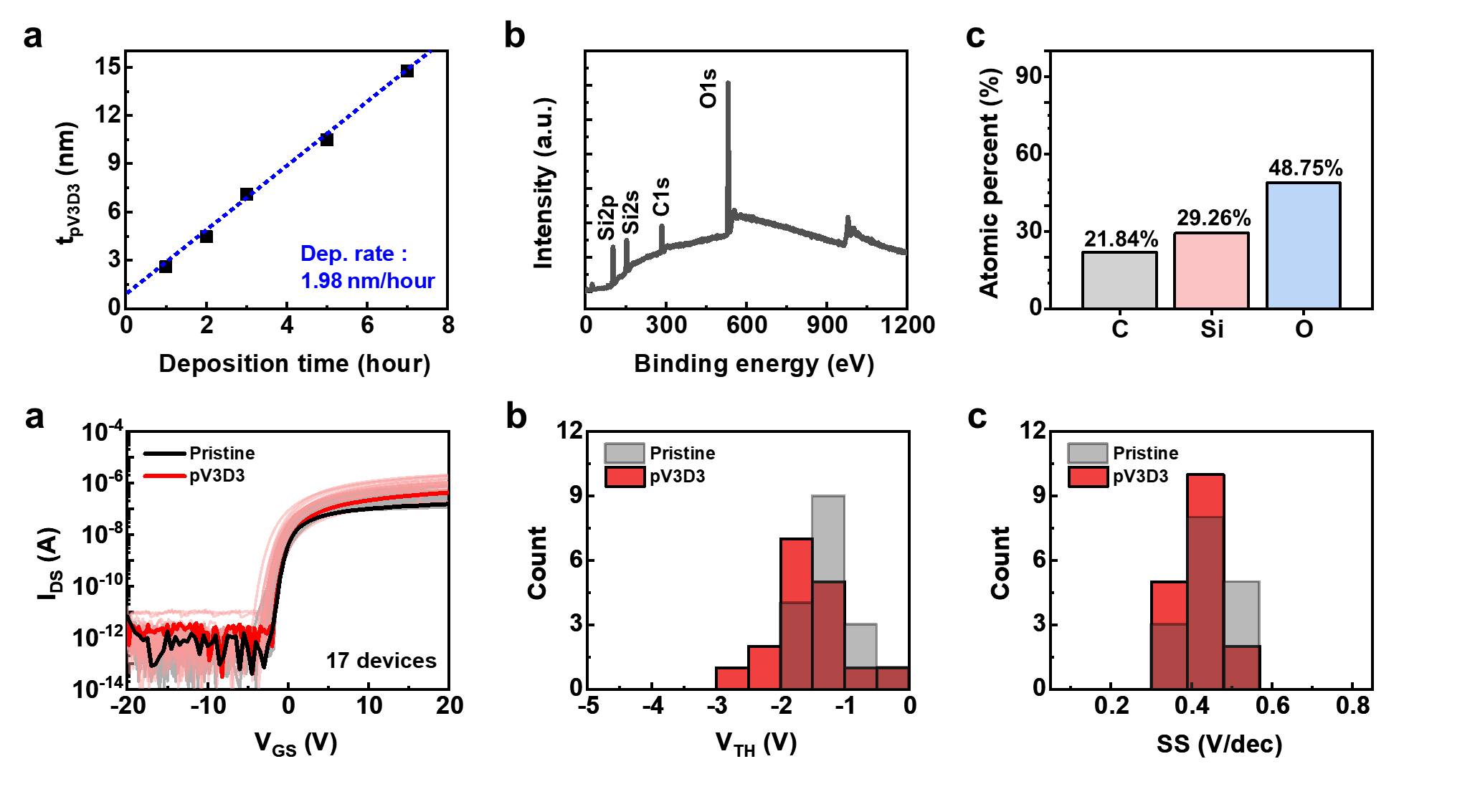
**

**Figure S4.** a) Transfer characteristics comparison between pristine MoS_2_ back-gate transistors and those after the deposition of 2 nm pV3D3 on MoS_2_. Statistical distributions of b) threshold voltage (*V*_TH_) and c) subthreshold slope (*SS*) for pristine MoS_2_ back-gate transistors and after the deposition of 2 nm pV3D3, showing an 8% enhanScement in average value of SS and a negligible negative shift in the average V_TH_​ by -0.35V after pV3D3 deposition on MoS_2_.


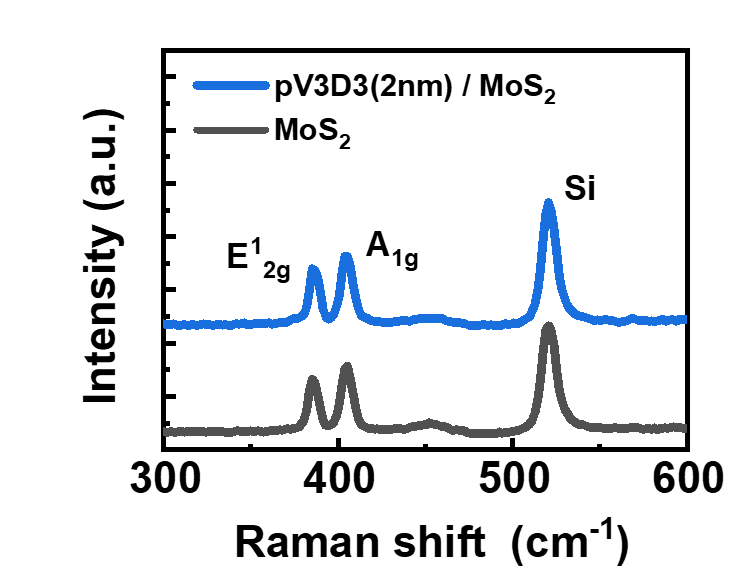


**Figure S5.** Comparison of Raman spectroscopy of MoS_2_ before and after deposition of iCVD based pV3D3 dielectric.

**
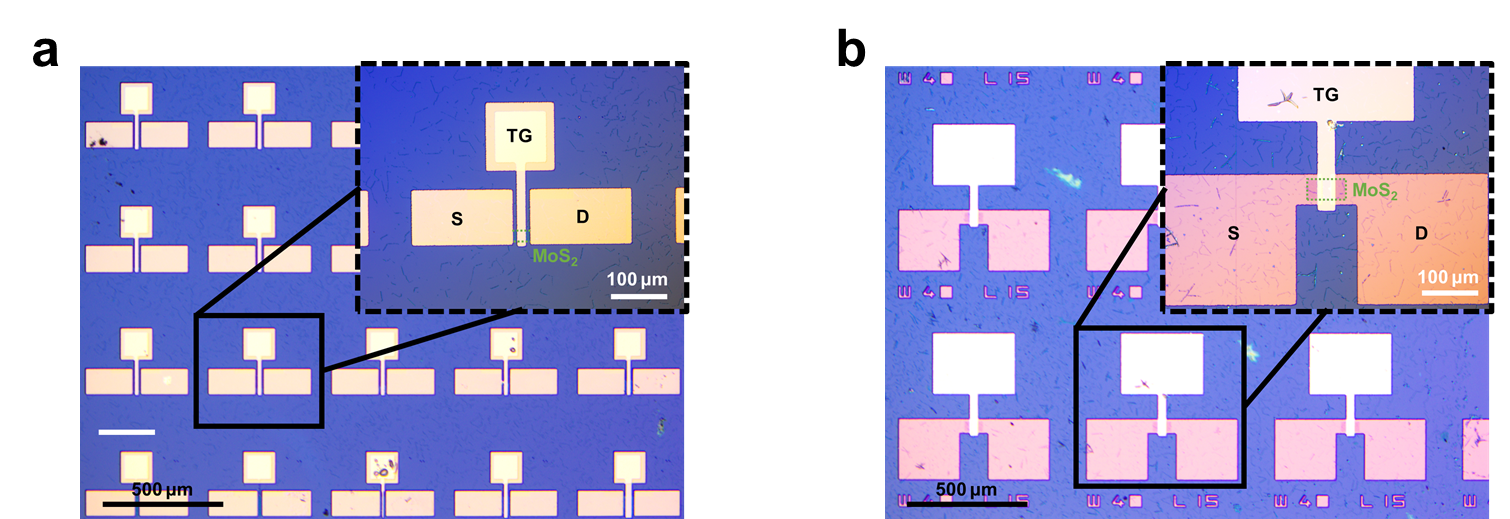
**

**Figure S6.** Optical microscope (OM) image of a) underlap-TG and b) overlap-TG structured MoS_2_ top-gate transistors.


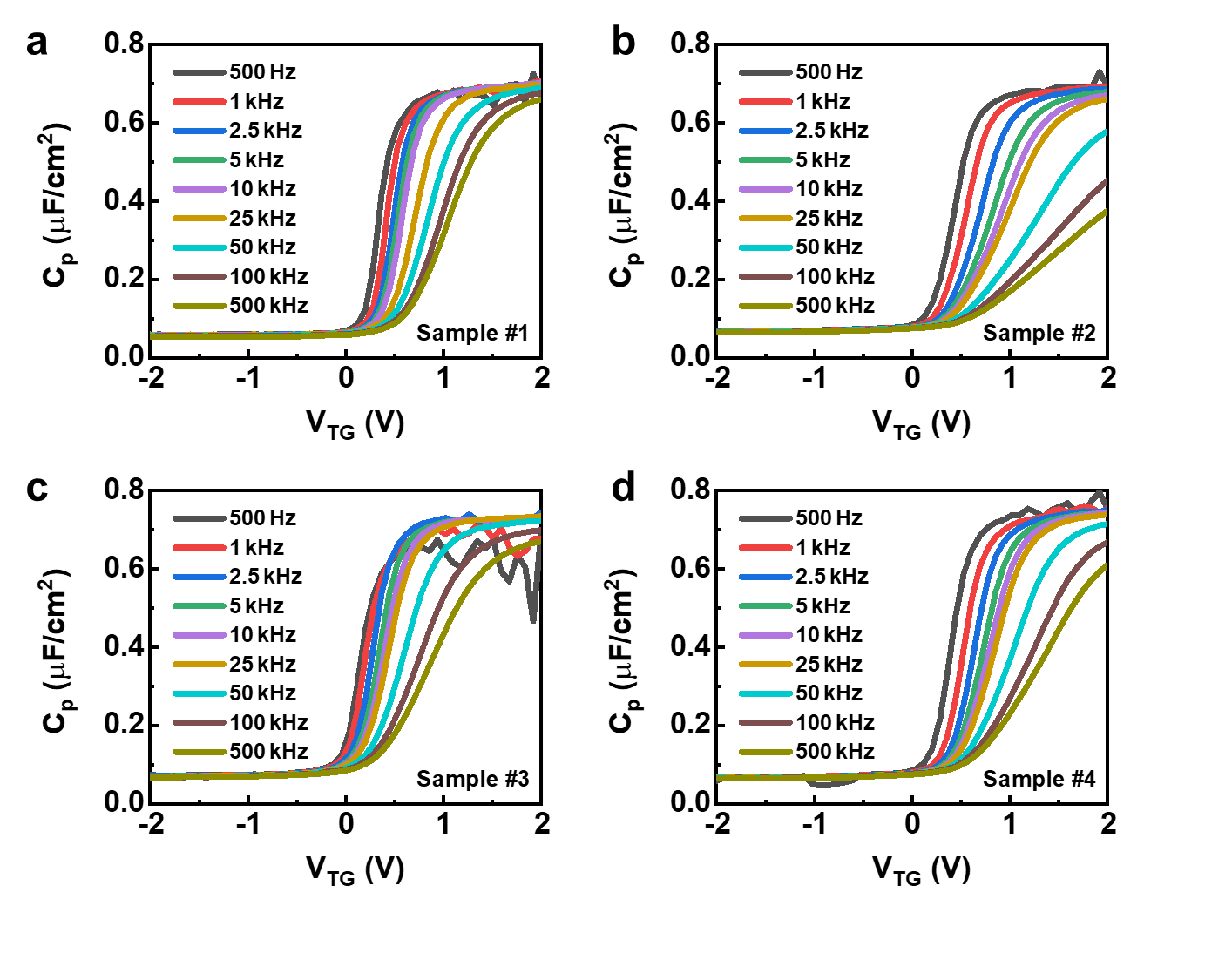


**Figure S7. (a–d)** Frequency-dependent capacitance–voltage (C–V) characteristics of MIS capacitors measured from 500 Hz to 500 kHz for four different devices incorporating pV3D3 as an inter-dielectric for top-gate insulator integration.

The interface trap capacitance (𝐶_𝑖𝑡_) can be calculated using the **Equation S1**.^[2]^

$$C_{it}=\left( \frac{1}{C_{LF}}-\frac{1}{C_{OX}} \right)^{-1}-\left( \frac{1}{C_{HF}}-\frac{1}{C_{OX}} \right)^{-1} (\boldsymbol{Equation} \boldsymbol{S}\boldsymbol{1})$$

Here, *C*_LF_ and *C*_HF_ correspond to the low-frequency (quasi-static) and high-frequency capacitances, respectively. The trap density derived from above claculation at depletion state is approximately 6.6×10^10^ cm^-2^e^-1^V^-1^ where *C*_LF_ and *C*_HF_ corresponding to the depletion capacitance of the device at 500 Hz and 500 kHz respectively.

**
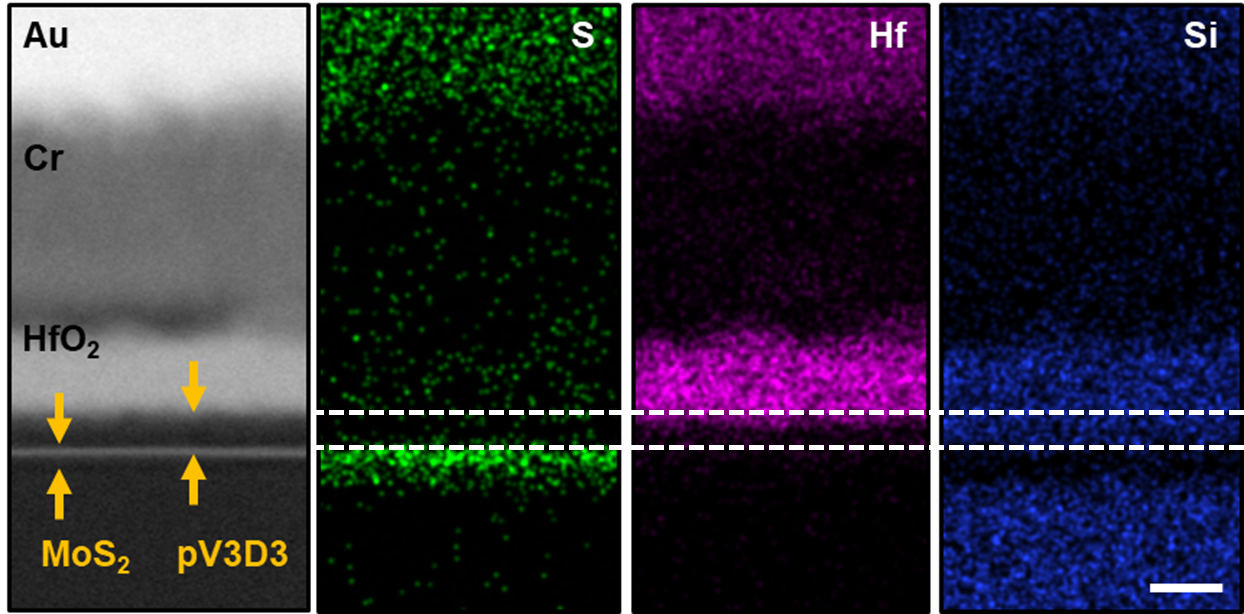
**

**Figure S8.** Cross-sectional dark-field (DF) STEM image of the pV3D3/HfO_2_/metal-gate (Cr/Au) stack deposited on a monolayer MoS_2_ and STEM–EDS elemental mapping of sulfur (S), hafnium (Hf), and Silicon (Si), illustrating the spatial distribution of the elements (Scale bar: 5nm).


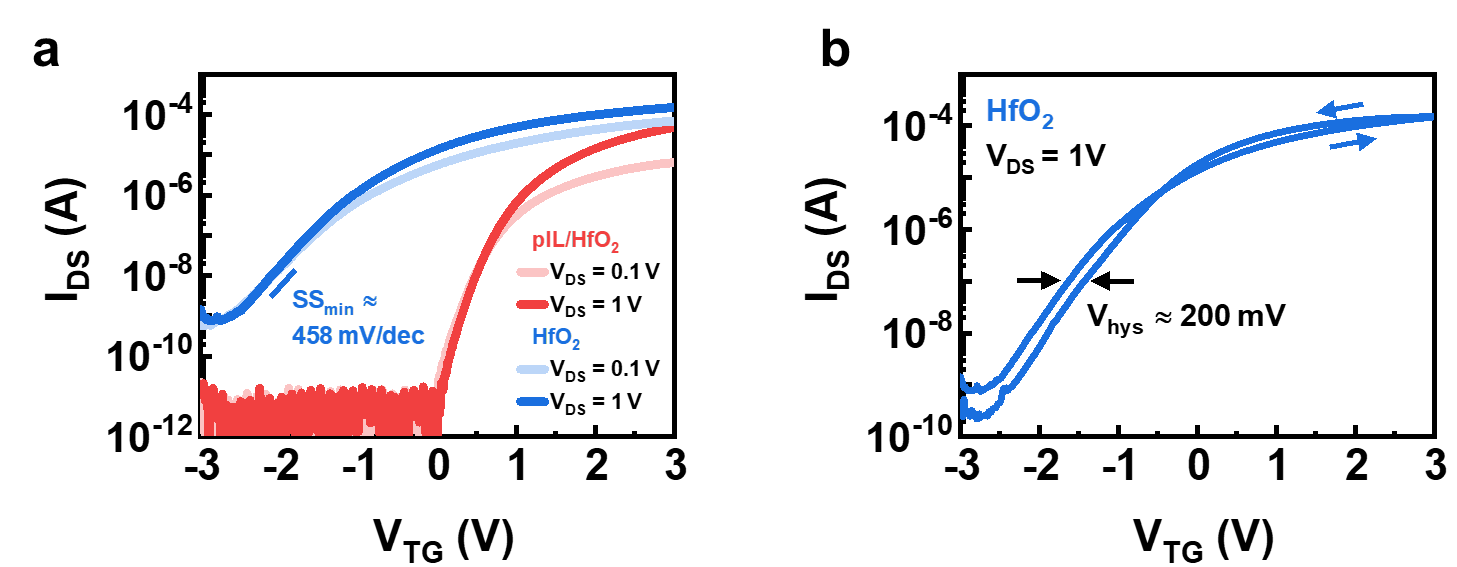


**Figure S9.** (a) Transfer characteristics of overlap top-gated MoS_2_ transistors with a 6 nm HfO_2_ gate dielectric, with and without a 2 nm pIL insertion layer, measured at *V*_DS_ of 0.1V and 1V. (b) Dual-sweep transfer curve of the HfO_2_-only device at *V*_DS_ = 1 V.


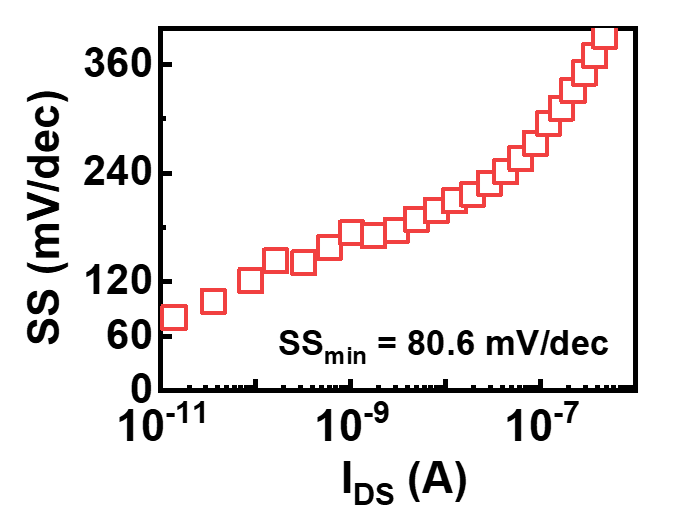


**Figure S10.** Subthreshold slope of overlap-TG MoS_2_ transistor (**Figure 4b**) as a function of drain current (*I*_DS_).


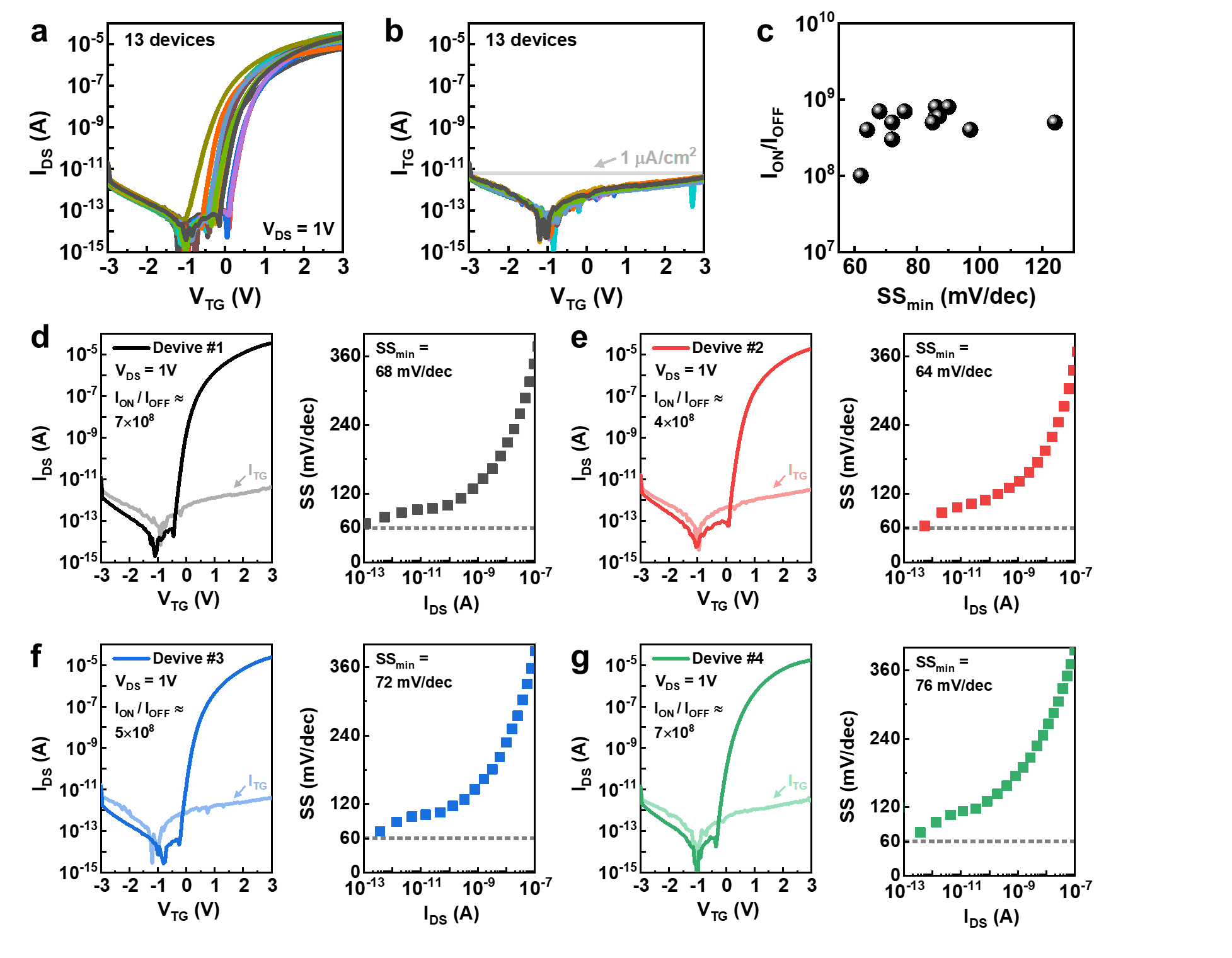


**Figure S11.** a) Transfer characteristics and b) gate leakage properties of 13 different overlap-TG MoS_2_ transistors in a single batch, measured using a high-resolution source measurement unit (SMU) and c) statistical distributions of *I*_ON_/*I*_OFF_ and *SS*_min_ of them. d) – g) Transfer characteristics, gate leakage current (*I*_TG_) property and *SS* as a function of drain current (I_DS_) for the representative 4 different best-performing overlap-TG MoS_2_ devices

To accurately assess the off-state current characteristics below 1 pA and thereby ensure the reliable extraction of potential *I*_ON_/*I*_OFF_ ratios and *SS* of our overlap-TG MoS_2_ transistors, we employed a high-resolution SMU (Keysight B1500A) to characterize the transfer and gate leakage properties of 13 different devices fabricated within a single batch. All devices exhibited *I*_TG_ densities below 2 μA/cm^2^ across the entire gate voltage range, confirming the robust insulating properties of the pIL/HfO_2_ dielectric stack on 2D MoS_2_. The devices consistently exhibited enhance mode behavior, with *I*_ON_/*I*_OFF_ ratios exceeding 10^8^. Notably, the best-performing device (**Figure S11e**.) demonstrated an *I*_ON_/*I*_OFF_ ratio exceeding 10^8^ along with a *SS*_min_ of 64 mV/dec.


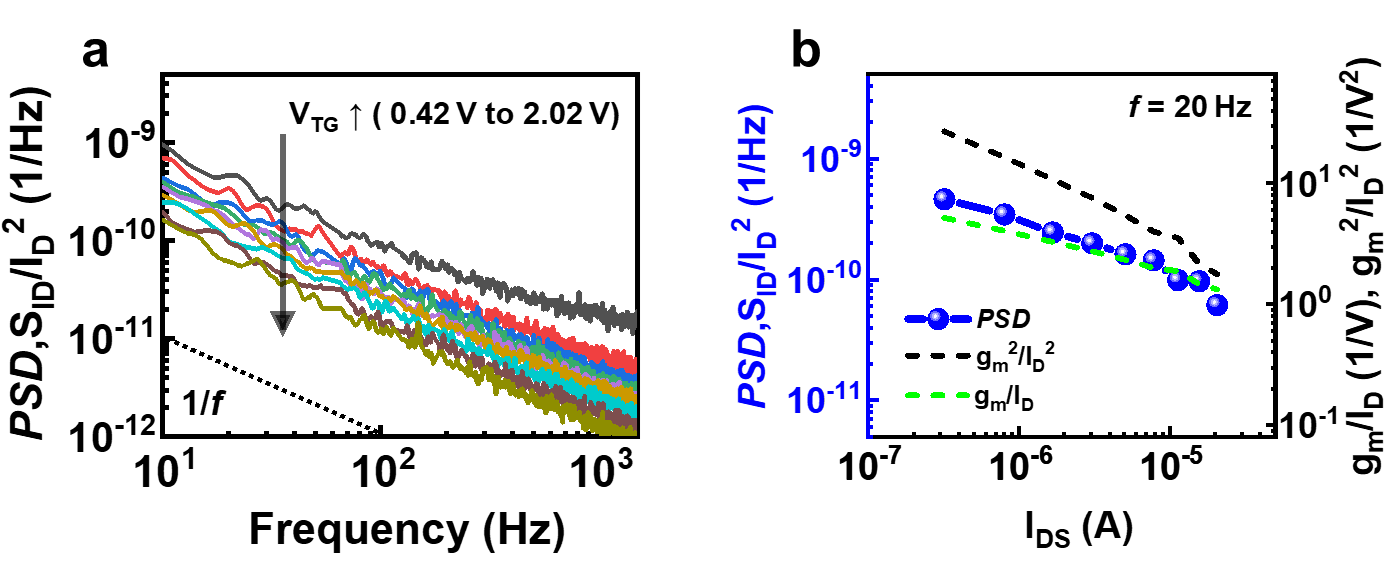


**Figure S12.** a) Noise power spectrum density at different *V*_TG_ from 0.42 to 2.02 V and *V*_DS_ = 1 V. where the dashed line exhibits the slope of 1/*f*. b) Normalized drain current spectral density as a function of I_DS_ with the fitting data of Hooge mobility fluctuation (HMF, dashed green line) and carrier number fluctuation (CNF, dashed black line).

Low-frequency noise (LFN) analysis was performed under ambient conditions to probe the charge transport characteristics and assess potential contributions from carrier trapping mechanisms as shown in **Figure S12a**. The measured drain current noise power spectral density (*S*_ID_) exhibits a typical 1/*f*^γ^ dependence, with γ ranging from 0.9 to 1, confirming 1/*f*-type behavior across the measured gate voltage and frequency range.

To elucidate the origin of the 1/*f* noise, two representative models were considered: the Hooge mobility fluctuation (HMF) model and the carrier number fluctuation (CNF) model. The HMF model attributes low-frequency noise to fluctuations in carrier mobility due to scattering processes in the channel region, predicting a linear dependence of the normalized noise power spectral density (PSD, *S*_ID_/*I*_D_²) on *g*_m_/*I*_D_ by the following equation where *q*, *α*_H_, *μ*, *V*_D_, and *L* are the elemental charge, Hooge parameter, mobility, drain voltage, and channel length, respectively.

$$\frac{S_{ID}}{{I_{D}}^{2}}= \frac{{{q\alpha}_{H}V}_{D}}{fL^{2}}\frac{1}{I_{D}} (\boldsymbol{Equation S}\mathbf{2})$$

Our experimental data showed agreement with this model, as evidenced by the linear correlation in **Figure S12b** and the extracted Hooge parameter (*α*_H_) was 0.036, which is comparable to previously reported values.^[10]^

Conversely, the CNF model relates the noise to fluctuations in carrier number due to trapping and de-trapping at interface or oxide trap sites, predicting a dependence of *S*_ID_/*I*_D_^2^ on (*g*_m_/*I*_D_)^2^ through the following equation where *g*_m_ and *S*_Vfb_ are the transconductance and flat band voltage spectral density, respectively.

$$\frac{S_{ID}}{{I_{D}}^{2}}= \left( \frac{g_{m}}{I_{D}} \right)^{2}S_{V_{fb}} (\boldsymbol{Equation S}\mathbf{3})$$

However, our measurements revealed no significant correlation with (*g*_m_/*I*_D_)^2^, as shown in **Figure S12b**, thereby ruling out CNF as the dominant noise mechanism. Those results suggest that carrier trapping does not play a significant role in the observed noise behavior but by the mobility fluctuations induced by carrier scattering near the channel.

**
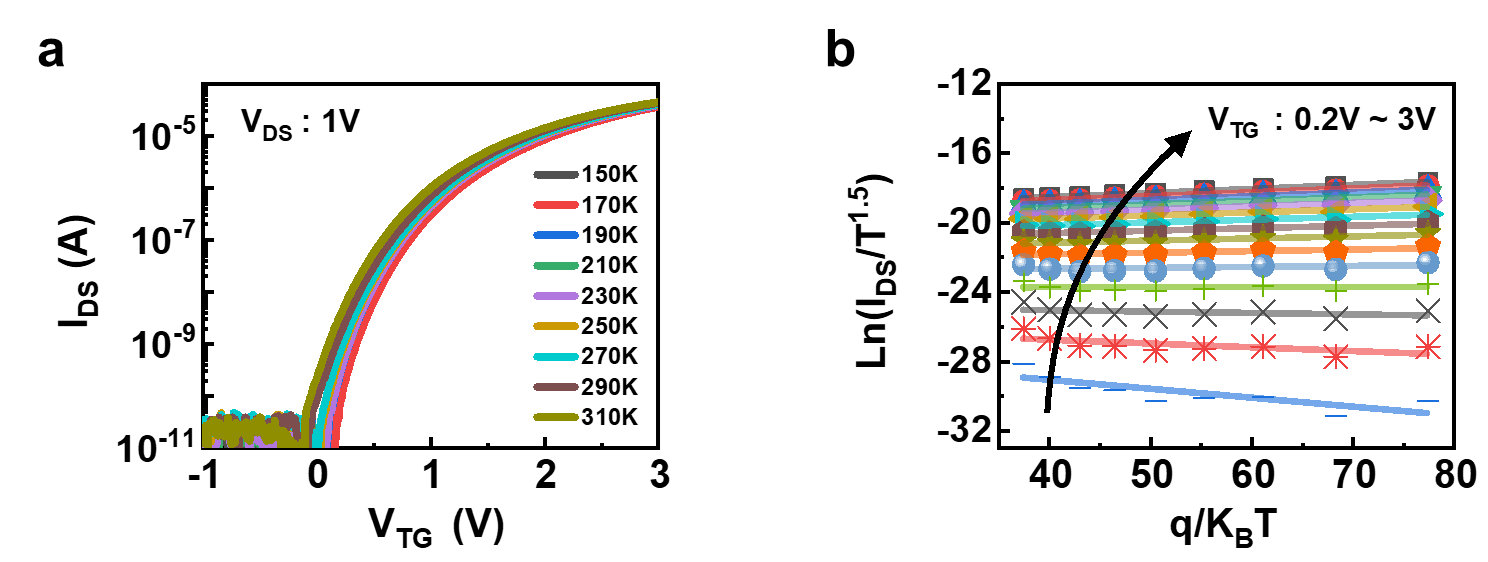
**

**Figure S13.** a) Transfer characteristics of overlap-TG MoS_2_ transistor for various temperature ranging from 150K to 310K, b) Arrhenius plots of the overlap-TG MoS_2_ transistor for various *V*_TG_ ranging from 0.2V to 3V with a bias step of 0.2V.

The Schottky barrier height (SBH, $\Phi_{SB}$) was extracted using the two-dimensional thermionic emission model (**Equation S4**), where *A*^*^ denotes the Richardson constant for 2D materials, m^⁎^ is the electron effective mass, *T* is the temperature, *k*_B_ is the Boltzmann constant, and *q* is the elementary charge.^[11]^

$$I_{DS}=A_{2D}^{*}T^{1.5} exp\left( -\frac{\Phi_{Eff}}{k_{B}T} \right) \left[ 1-exp\left( -\frac{V_{DS}}{k_{B}T} \right) \right] (\boldsymbol{Equation S}\boldsymbol{4})$$

Temperature-dependent transfer characteristics of the overlap-TG MoS_2_ transistor were measured at a fixed *V*_DS_ of 1 V across various gate voltages (**Figure S13a**), and the corresponding Arrhenius plots are presented in **Figure S13b**. The slope of each plot reflects the effective SBH ($\Phi_{Eff}$) at a given *V*_TG_. By extrapolating $\Phi_{Eff}$ as a function of *V*_TG_, the $\Phi_{SB}$ was extracted, as shown in **Figure 4d**.


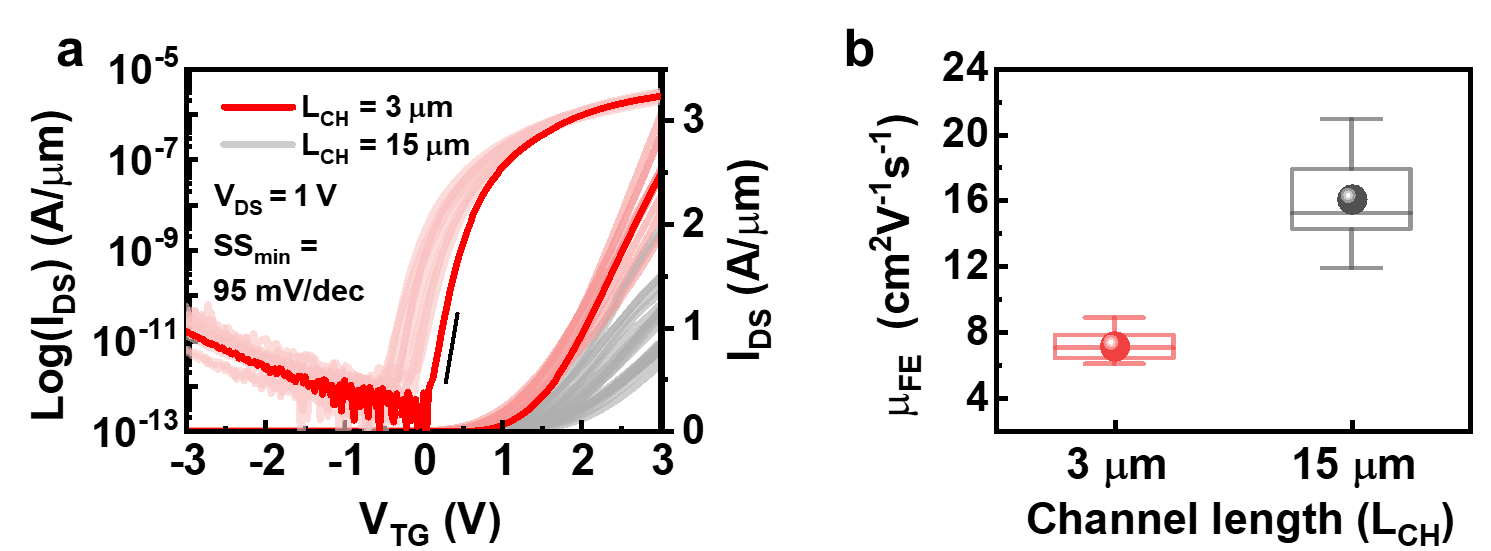


**Figure S14.** a) Transfer characteristics of overlap-TG MoS_2_ transistors with channel lengths (*L*_CH_) of 3 μm and 15 μm, incorporating a pV3D3 inter-dielectric layer measured at *V*_DS_ of 1V b) Comparison of extracted field-effect mobility.

**
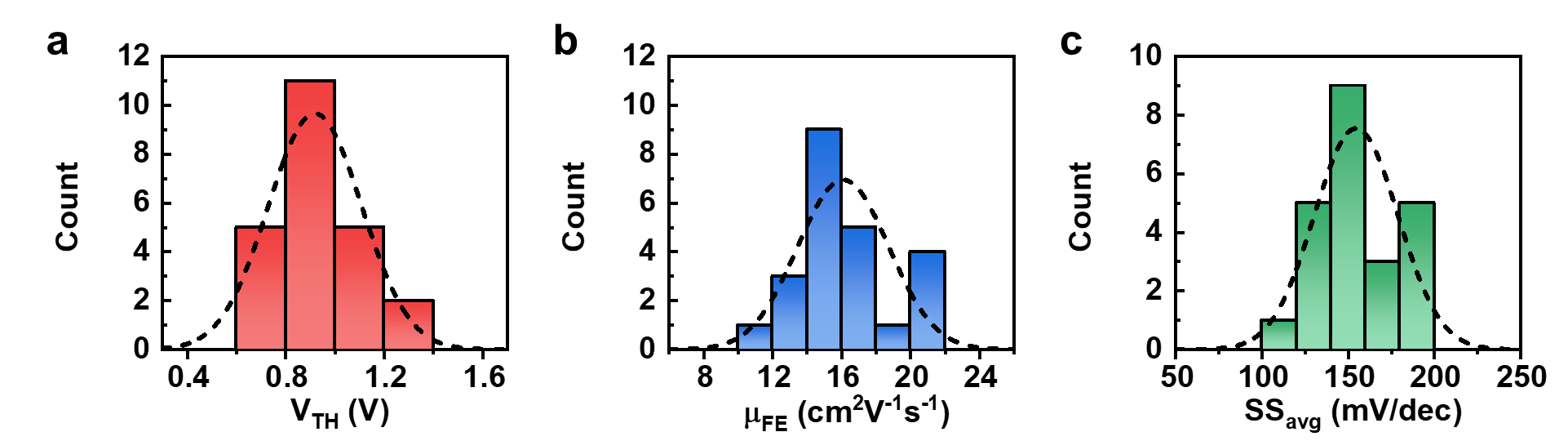
**

**Figure S15.** Statistical distributions of a) *V*_TH_, b) field effect mobility ($\mu_{\mathrm{FE}}$) and c) 3-order average subthreshold slope (*SS*_avg_) for 23 devices in a single batch.

**Figure S15** presents the statistical distribution of electrical performance across 23 devices within a single batch. As shown in **Figure S15a** and **Figure** **S15b**, all devices exhibit positive *V*_TH_ with average *V*_TH_ value of 0.9V, operating in enhancement mode, with a maximum and average $\mu_{\mathrm{FE}}$ of 20 cm^2^V^-1^s^-1^ and 16 cm^2^V^-1^s^-1^ respectively and the distribution of *SS*_avg_ extracted over the current range of 10 pA to 10 nA is shown in **Figure S15c**.

**
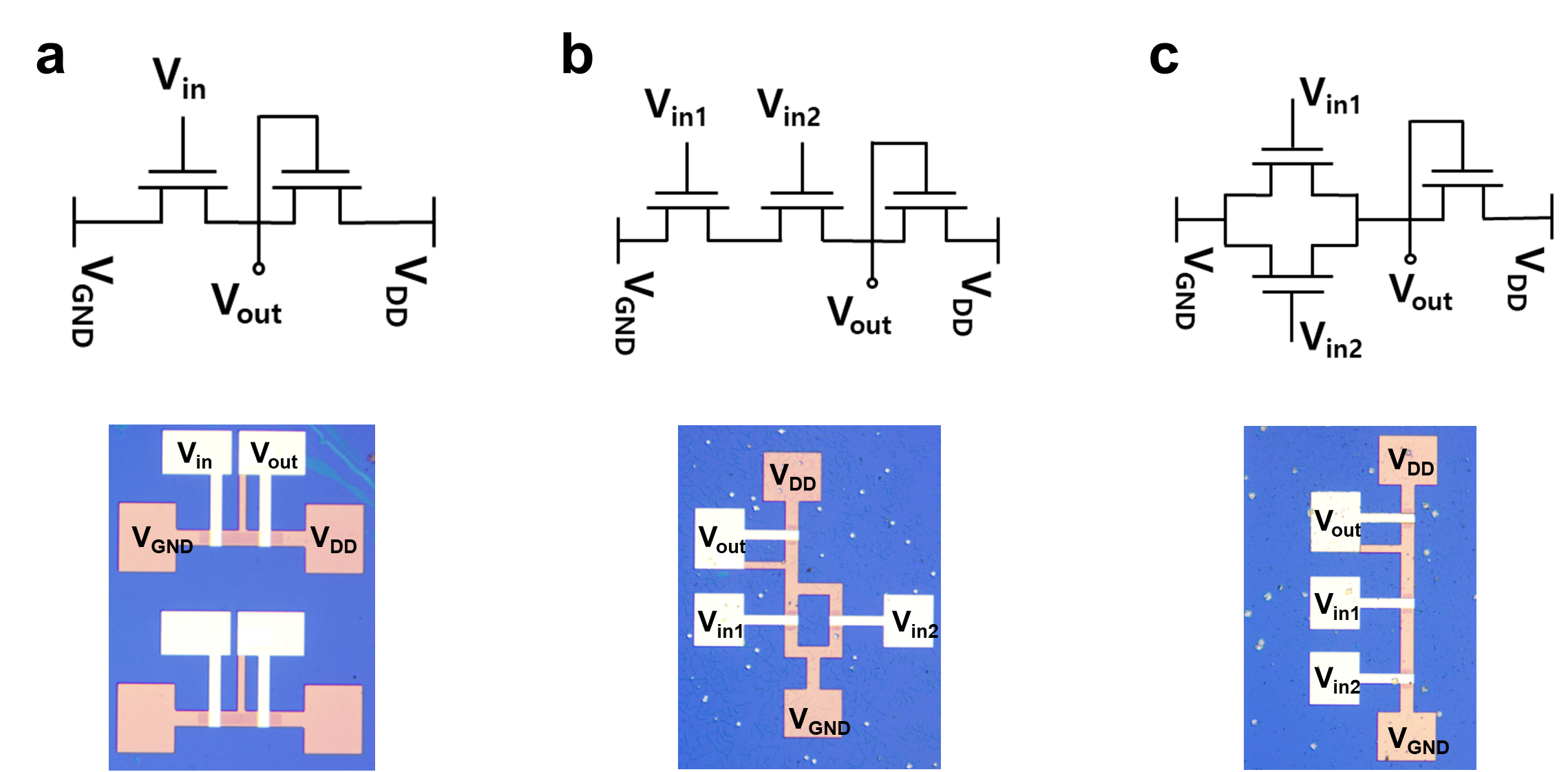
**

**Figure S16.** Optical microscope (OM) image and schematic equivalent circuit illustration of the fabricated logic gates: a) Inverter (NOT), b) NAND and c) NOR logic gates

**
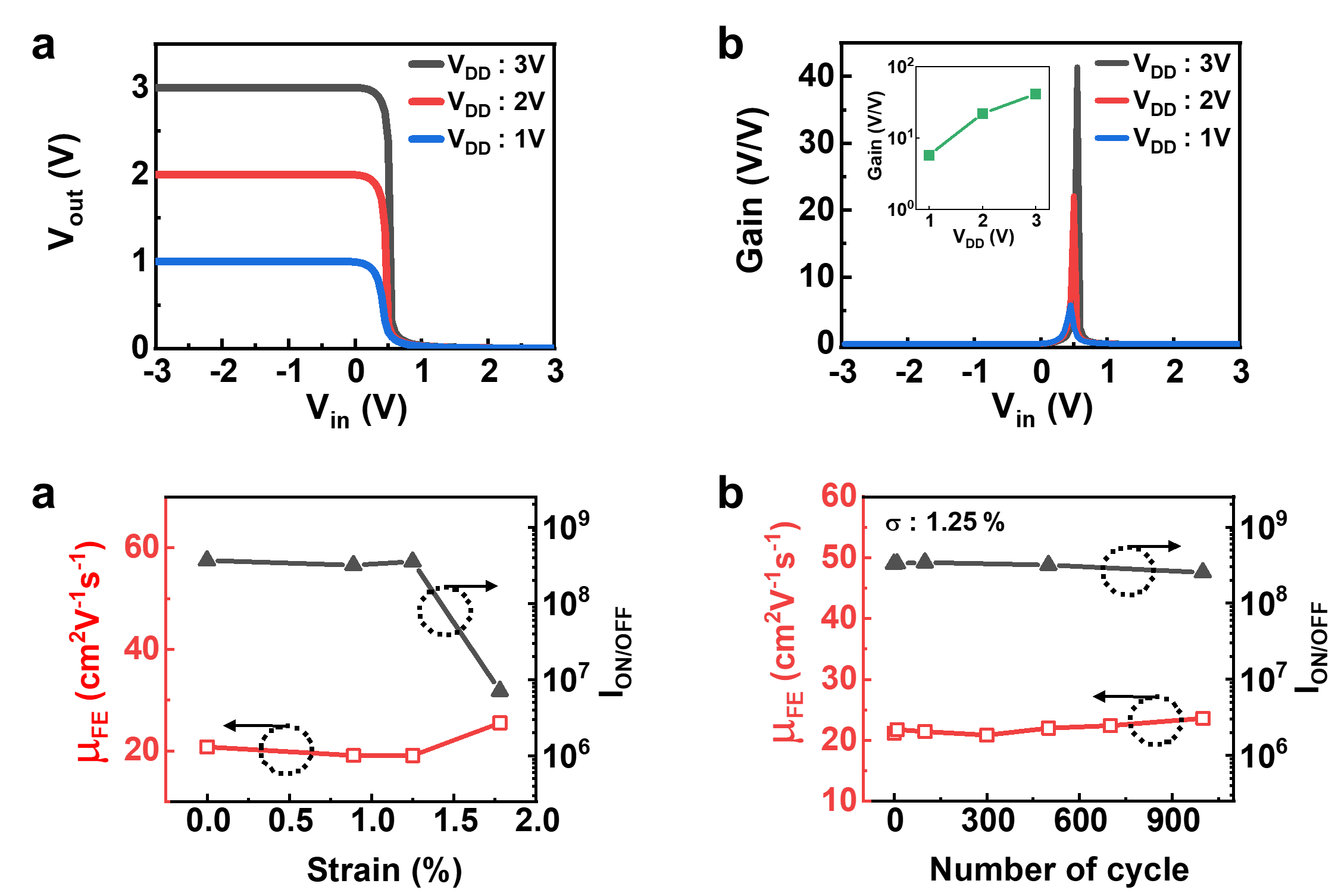
**

**Figure S17.** a) The voltage transfer characteristics as a function of input voltage (*V*_IN_) under various supply voltage (*V*_DD_) from 1V to 3V. b) The corresponding voltage gain characteristics are shown, with the inset highlighting the gain for each supply voltage.


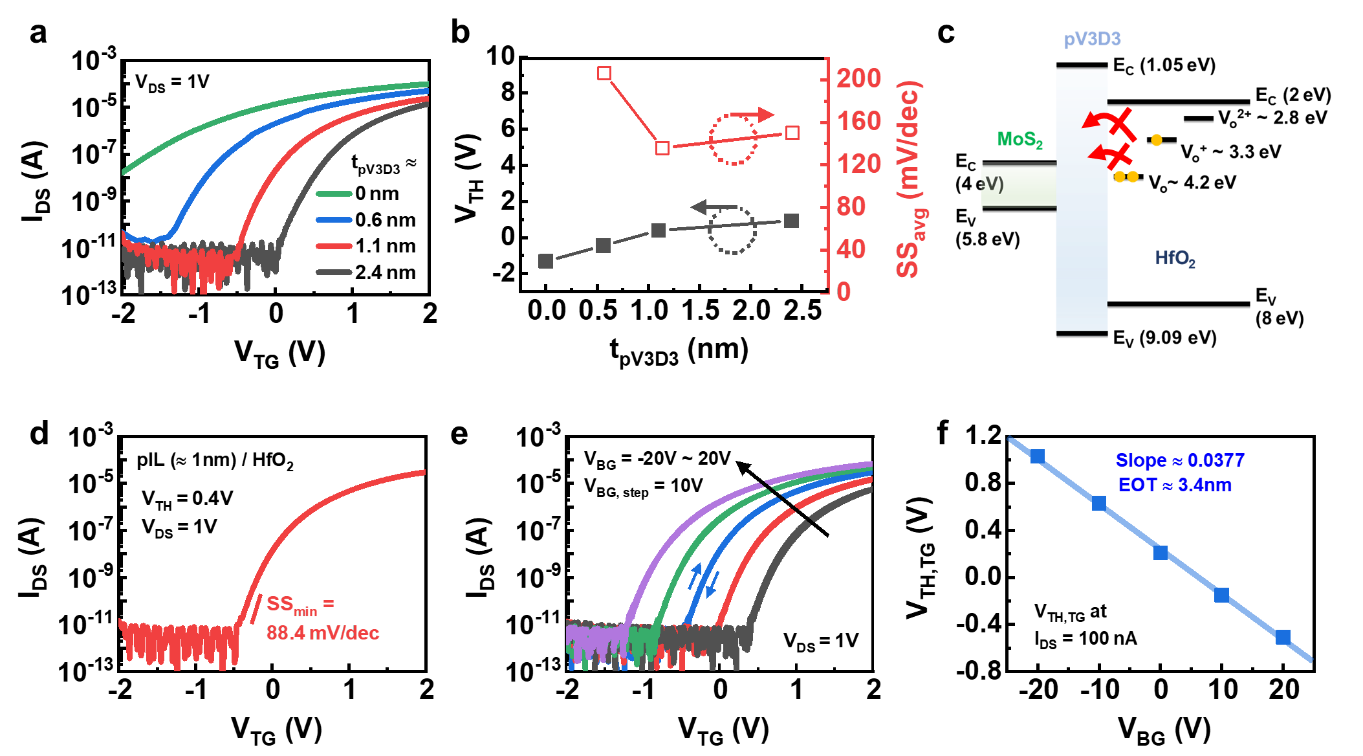


**Figure S18.** a) Transfer characteristics of overlap top-gate MoS_2_ transistors with various pIL thickness. b) Variation in *V*_TH_ and *SS*_avg_ with pIL thickness variation. c) Schematic band diagram illustrating the suppression of charge diffusion induced from HfO_2_ defect states by the pIL. d) Transfer characteristics of the best-performing device incorporating about 1 nm pIL interlayer. e) Transfer characteristics under varying back-gate biases (*V*_BG_ ranging from -20 V to +20 V). f) *V*_TH_ modulation of the top-gate transistor as a function of applied various *V*_BG_.

As shown in **Figure S18a** and **Figure S18b**, reducing the pV3D3 thickness below 2 nm results in a negative shift in the *V*_TH_ and a degradation in *SS*_avg_, particularly when the thickness approaches below sub-1 nm range. This degradation is attributed to the diminished ability of ultrathin pV3D3 layers to suppress electron tunneling **(Figure S18c**) and electron trapping from the overlying HfO_2_ induced intrinsic defect states.^[12]^

Nevertheless we experimentally confirmed that the MoS_2_ top-gate transistor can still exhibit excellent performance even with a pV3D3 thickness of approximately 1 nm, which contributes to EOT reduction and, consequently, enables lower operating voltage. **Figure S18d** shows the best-performance device adopting ~ 1nm pV3D3 interlayer. Compared to the device without the interlayer (**Figure S9b**), the insertion of about 1 nm pV3D3 effectively shifts *V*_TH_ toward positive values (*V*_TH_ ≈ 0.4 V), suppressing uncontrollable HfO_2_ defect related charge transfer doping. It also significantly improves the interface quality, achieving a *SS*_min_ of 88.4 mV/dec and hysteresis of 15 mV at *V*_BG_ of 0V.

**Figure S18e** and **Figure S18f** further present the transfer characteristics of the device under varying *V*_BG_. The top-gate transistor shows a linear modulation of *V*_TH, TG_ with V_BG_, without degradation in SS or hysteresis, confirming effective electrostatic coupling through the dielectric stack. From the *V*_TH, TG_ shift with V_BG_, we extracted a coupling ratio between the capacitance of the back-gate insulator (*C*_BG_) and the top-gate insulator stack (*C*_TG_) that corresponds to EOT of 3.4 nm. This EOT value is consistent with a physical pV3D3 thickness of approximately 1.2 nm, validating that the practical lower limit for the interlayer thickness, considering both EOT scaling and *V*_TH_ controllability, is around 1 nm.

**
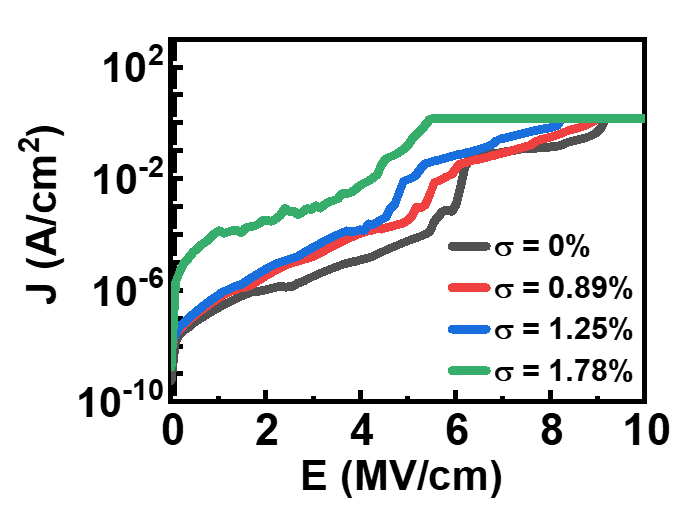
**

**Figure S19.** Leakage current density (*J*) characteristics as a function of applied electric field of the pV3D3/HfO_2_ gate dielectric stack on polyimide (PI) substrate under various tensile strain condition ranging from 0% to 1.78%.

**
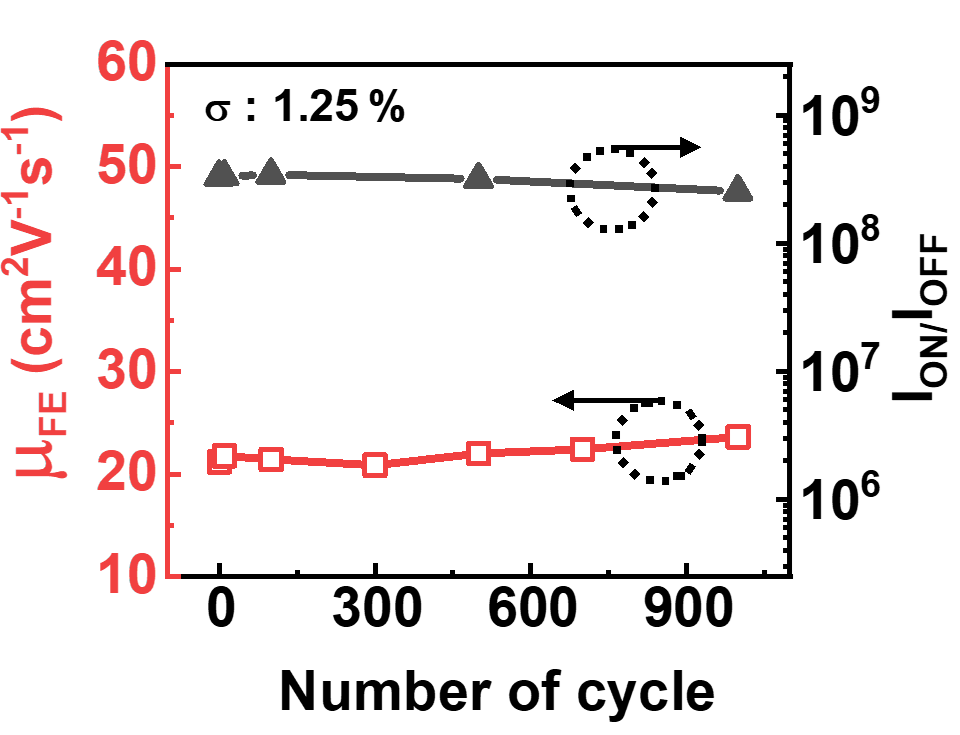
**

**Figure S20.** The $\mu_{\mathrm{FE}}$ and *I*_ON_/*I*_OFF_ ratio variations during 10^3^ cycling test under 1.25% strain.

**
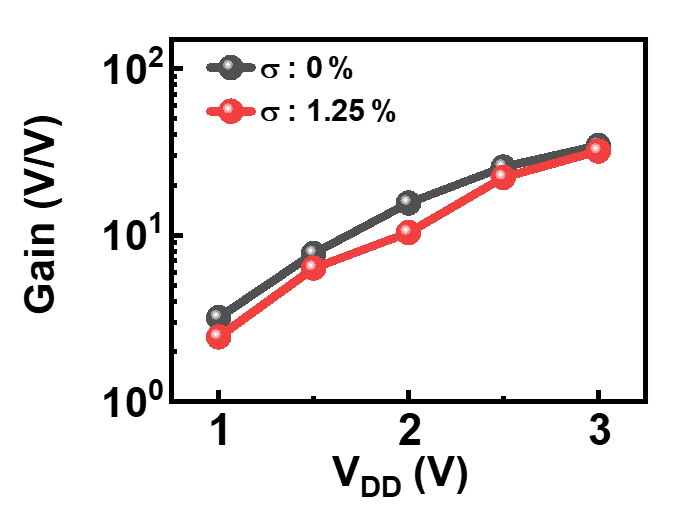
**

**Figure S21.** Variation in voltage gain across different *V*_DD_ values (*V*_DD_: 1 V to 3 V in 0.5 V increments) under 0% and 1.25% tensile strain conditions.

**References**

[1] W. Li, J. Zhou, S. Cai, Z. Yu, J. Zhang, N. Fang, T. Li, Y. Wu, T. Chen, X. Xie, *Nature Electronics* **2019**, 2, 563.

[2] L. Wang, P. Wang, J. Huang, B. Peng, C. Jia, Q. Qian, J. Zhou, D. Xu, Y. Huang, X. Duan, *Nature Nanotechnology* **2022**, 17, 1206.

[3] Y. Xu, T. Liu, K. Liu, Y. Zhao, L. Liu, P. Li, A. Nie, L. Liu, J. Yu, X. Feng, *Nature Materials* **2023**, 22, 1078.

[4] Z. Lu, Y. Chen, W. Dang, L. Kong, Q. Tao, L. Ma, D. Lu, L. Liu, W. Li, Z. Li, *Nature communications* **2023**, 14, 2340.

[5] C. Sheng, X. Wang, X. Dong, Y. Hu, Y. Zhu, D. Wang, S. Gou, Q. Sun, Z. Zhang, J. Zhang, *Advanced Functional Materials* **2024**, 34, 2400008.

[6] H. Uchiyama, K. Maruyama, E. Chen, T. Nishimura, K. Nagashio, *Small* **2023**, 19, 2207394.

[7] J. Ma, X. Chen, X. Wang, J. Bian, L. Tong, H. Chen, X. Guo, Y. Xia, X. Zhang, Z. Xu, *ACS Applied Materials & Interfaces* **2022**, 14, 11610.

[8] M. J. Liu, W. J. Lan, C. S. Huang, C. Z. Chen, R. H. Cyu, P. A. L. Sino, Y. L. Yang, P. W. Chiu, F. C. Chuang, C. H. Shen, *Small* **2024**, 20, 2307728.

[9] D. Zeng, Z. Zhang, Z. Xue, M. Zhang, P. K. Chu, Y. Mei, Z. Tian, Z. Di, *Nature* **2024**, 632, 788.

[10] a)G. H. Shin, G.-B. Lee, E.-S. An, C. Park, H. J. Jin, K. J. Lee, D. S. Oh, J. S. Kim, Y.-K. Choi, S.-Y. Choi, *ACS Applied Materials & Interfaces* **2020**, 12, 5106; b)V. K. Sangwan, H. N. Arnold, D. Jariwala, T. J. Marks, L. J. Lauhon, M. C. Hersam, *Nano letters* **2013**, 13, 4351; c)J.-K. Kim, Y. Song, T.-Y. Kim, K. Cho, J. Pak, B. Y. Choi, J. Shin, S. Chung, T. Lee, *Nanotechnology* **2017**, 28, 47LT01; d)Y. Wang, X. Luo, N. Zhang, M. R. Laskar, L. Ma, Y. Wu, S. Rajan, W. Lu, presented at *82nd ARFTG Microwave Measurement Conference*, **2013**.

[11] a)P.-C. Shen, C. Su, Y. Lin, A.-S. Chou, C.-C. Cheng, J.-H. Park, M.-H. Chiu, A.-Y. Lu, H.-L. Tang, M. M. Tavakoli, *Nature* **2021**, 593, 211; b)M. Kang, W. Hong, I. Lee, S. Park, C. Park, S. Bae, H. Lim, S.-Y. Choi, *ACS Applied Materials & Interfaces* **2024**, 16, 43849.

[12] K. Xiong, J. Robertson, M. Gibson, S. Clark, *Applied physics letters* **2005**, 87.
